# Translational difficulties in establishing a pharmacologically induced neurovascular uncoupling model in rats

**DOI:** 10.1038/s41598-026-58244-0

**Published:** 2026-06-17

**Authors:** Bence Tamás Varga, Aliz Judit Ernyey, Brigitta Tekla Tajti, Attila Gáspár, Zsuzsanna Demeter, Levente Kollár, Péter Kovács, Mihály Albert, Alán Alpár, István Gyertyán

**Affiliations:** 1 Department of Pharmacology and Pharmacotherapy, Semmelweis University, Budapest, Hungary; 2 Center for Pharmacology and Drug Research & Development, Semmelweis University, Budapest, Hungary; 3 Medicinal Chemistry Research Group and Drug Innovation Centre, HUN-REN Research Centre for Natural Science, Budapest, Hungary; 4 CEVA-Phylaxia Ltd. (Ceva Sante Animale), Budapest, Hungary; 5 Department of Anatomy, Histology and Embryology, Semmelweis University, Budapest, Hungary

**Keywords:** Neurovascular coupling, Cognitive decline, Animal model, MS-PPOH, L-NAME, Indomethacin, Diseases, Drug discovery, Medical research, Neurology, Neuroscience

## Abstract

Neurovascular uncoupling (NVU) contributes to neurological disorders like Alzheimer’s disease. While a mouse NVU model exists, a reliable rat model critical for cognitive research remains underdeveloped. To address this methodological gap, we investigated a pharmacological approach in rats using the same drugs (N-(methylsulfonyl)-2-(2-propynyloxy)-benzenehexanamide (MS-PPOH), L-NG-nitroarginine methyl ester (L-NAME), indomethacin) that proved to be efficacious in mice. The compounds were formulated as a cocktail solution and administered intraperitoneally for 13 days to aged, cognitively experienced Long-Evans rats. Our goal was to induce NVU while minimizing adverse systemic effects seen previously (e.g., hypertension, intestinal ulceration). The treatment induced only a modest (28%, non-significant) reduction in cerebral hyperaemia, with decreased prostaglandin E2 levels but unchanged 11,12-epoxyeicosatrienoic acid concentration in the brain. Cognitive effects were limited—transient impairment in the 5-choice task but no changes in spontaneous alternation, visual discrimination, cooperation, or motor learning. Significant adverse effects emerged: reduced food intake, weight loss, gastrointestinal malaise, and moderate renal toxicity. Our findings specifically highlight the challenges of achieving sufficient and symptomatically apparent NVU while minimizing systemic toxicity. While partial NVU occurred, this polypharmacy approach had major limitations. A reliable, industrially applicable rat NVU model remains urgently needed to accelerate antidementia drug development.

**Supplementary Information:**

The online version contains supplementary material available at https://doi.org/10.1038/s41598-026-58244-0.

## Introduction

Neurovascular coupling (NVC) is a physiological mechanism that ensures precise regulation of cerebral blood flow (CBF) in response to the metabolic demands of active neural circuits^1–3^. The neurovascular unit—neurons, astrocytes, pericytes, and endothelial cells—orchestrates this process^1,2,4^. Astrocytes release vasoactive factors such as nitric oxide (NO), epoxyeicosatrienoic acids (EETs), and prostaglandins, which dilate blood vessels^1,5–8^. Pericytes regulate capillary diameter and are influenced by EETs^3,9^. Endothelial cells release NO and EETs, and EETs also help maintain blood–brain barrier integrity^6,7,10,11^. Potassium dynamics, including ATP-sensitive and Kir2.1 channels, contribute to NVC by propagating hyperpolarizing signals to upstream arterioles^12–14^.

Disruptions in NVC, termed neurovascular uncoupling (NVU), are implicated in a wide range of neurological disorders, including Alzheimer’s disease (AD), stroke, and age-related cognitive decline^2,15,16^. The same impairment is also observed in hypertension and diabetes^17–19^, where oxidative stress plays a central role. Specifically, stress-induced reduction of NO bioavailability directly leads to NVC uncoupling^20^. Importantly, NVU is not only a consequence but also a driver of pathology. In AD models, NVU precedes cognitive deficits^21^. Thus, preserving NVC is a promising therapeutic strategy, and reliable animal models of NVU are essential for preclinical research, particularly in cognitive disorders.

Our research group has elaborated a rat cognitive test battery for putative cognitive enhancer compounds with the aim to achieve improved predictive power^22–26^. In our -cognitive testing paradigm subjects are experienced rats trained in several cognitive tasks modelling various human cognitive domains, thereby mimicking the “baseline” knowledge level of human patients. Furthermore, this population allows the simultaneous measurement and longitudinal follow-up of the efficacy of a given impairing method on the various cognitive functions. The impairing method is a translationally critical factor as it should bear the construct validity of the induced cognitive deficit as a model of the clinical symptoms. The predictive power of the frequently used transmitter-specific pharmacological impairments (e.g. scopolamine, MK-801) has been criticized in the literature^27,28^) and we also obtained incoherent results with them^29–31^. As neurovascular uncoupling bears proven relevance to human cognitive decline, developing a pharmacologically induced rat NVU model and integrating it to our test system held the promise to establish a highly relevant model of age- and dementia-related cognitive deficits. Pharmacologically induced neurovascular uncoupling with subsequent neurological and cognitive defects was described in mice^32^, however, no similar procedure was reported in rats. So, we set out to transfer the published mouse model to rats.

Tarantini et al.^32^ induced NVU with the following 7 days long pharmacological treatment:

- N-(methylsulfonyl)-2-(2-propynyloxy)-benzenehexanamide (MS-PPOH), a specific inhibitor of epoxyeicosatrienoic acid (EET)-producing epoxigenases; 20 mg/kg/day applied via subcutaneously implanted Alzet osmotic minipump;
- L-NG-nitroarginine methyl ester (L-NAME), a NO synthase inhibitor, 100 mg/kg/day dissolved in drinking water;
- Indomethacin, a nonselective inhibitor of cyclooxygenases, 7.5 mg/kg per day, *per os*.

The above pharmacological treatment caused circa 2/3 decrease in the evoked cerebral functional hyperaemia and impaired learning performance in assays of spatial memory, recognition memory and motor coordination^32^.

In our previous study carried out in Wistar rats^9^ we applied the same three compounds but with reduced doses tailored to rats (MS-PPOH: 10 mg/kg/day, L-NAME: 20 mg/kg/day, indomethacin: 2 mg/kg/day) and administering them intraperitoneally (ip.) in a single cocktail solution. We established these doses after pilot dose-finding experiments aiming to ensure the tolerability of the pharmacological treatment while maintaining efficacy. We found impaired NVC, but the animals only showed marginal performance differences in response to treatment in cognitive tests. Furthermore, the treatment was nonetheless accompanied by undesirable side effects, such as significant increase in blood pressure and small intestine ulceration^9^.

The partial success of our first attempt prompted us to continue refining the methodology and we conducted a series of experiments, the results of which are presented in this paper. Our objectives were to: (i) establish NVU-inducing doses without undesirable side effects, (ii) identify cognitive assays sensitive to NVU, and (iii) validate the model in a translationally relevant population, namely, in aged, experienced rats.

## Methods

### General overview of the experiments performed

#### Experiment 1 (Exp 1)—Dose finding in young naïve Wistar rats

We halved the dosages of the treatment components used in our preliminary study to avoid the previously experienced toxic effects of the “cocktail” and introduced some new cognitive tests. Here we used young naïve Wistar rats.

#### Experiment 2 (Exp 2)—Cognitive effects of the uncoupling cocktail in aged, experienced Long-Evans rats

While further streamlining the dosages we used aged (27,5 months old), experienced male Long-Evans rats as subjects, the “default” strain in our cognitive test system. We introduced the animals to the NVU treatment, to see if their acquired knowledge shows impairment in response to it.

#### Experiment 3 (Exp 3)—Effects of MS-PPOH in young naïve rats

We wished to test the sole effects of MS-PPOH. Subjects were young, naïve Long-Evans rats.

#### Experiment 4 (Exp 4)—Conditioned taste aversion (CTA) test with the cocktail in aged rats

Adverse events in Exp 2, namely, increased amount of leftover food and rapid weight loss observed during the treatment raised concern about a possible gastrointestinal malaise effect caused by the cocktail in aged rats. To check this assumption, we performed a conditioned taste aversion (CTA) experiment with the NVU cocktail. Aged, male Wistar rats were available for this purpose.

The design of the NVU experiments (Exp 1–3) followed the same scheme. It involved a drug-treated and a vehicle-treated group and consisted of three phases: 1. baseline measurements, 2. treatment period with continued testing of the animals, 3. terminal surgery for neurovascular coupling measurement followed by ex vivo assays. Because of the limited throughput of terminal surgery, the animals were conducted through the experiment in cohorts of four.

Treatment of animals was done by separate people from those who performed the cognitive assays, and physiological measurements. The latter were unaware of what were the animals treated with (blinding).

All procedures carried out on animals were authorized by the regional animal health authority in Hungary (Pest County Government Office, resolution number PE/EA/785–5/2019 and PE/EA/00,808-7/2021 and conformed to the Hungarian welfare legislation and the EU 63/2010 Directive on the protection of animals used for scientific purposes. All procedures were carried out in accordance with the ARRIVE guidelines and the principles of the 3Rs (Replacement, Reduction, and Refinement).

### Housing of animals

Animals were housed in polycarbonate cages (1,376 cm^2^ area, 16 cm height) under reversed light–dark cycle (light phase from 4 pm to 4 am). The room they were kept in had an average temperature of 20–23 °C and humidity of 60–75%. Drinking water was available ad libitum. Cardboard tubes and aspen bricks were available as environmental enrichment. Animals were intensively handled before and during the experiments. Housing density and food access (commercial pellet rat feed (R/MZ + H, SSniff Spezialdiäten GmbH, Soest, Germany) varied by experiment (*see* below).

### Exp 1: dose finding in young naïve Wistar rats

For this study, we used 17 male Hannover Wistar rats (TOXI-COOP, Budapest, Hungary) weighing 155–215 g and being 2 months of age at the start of the experiment. Four animals were kept in a cage, except one cage containing five. Food was available ad libitum prior to treatment but restricted during treatment (15 g/rat supplied at least 2 h before the end of the dark phase).

### Treatment

For inducing NVU the following drugs were used: 2.5 mg/kg MS-PPOH, 0.5 mg/kg indomethacin, 5 mg/kg L-NAME, injected ip. for 9 consecutive days, twice per day (at 6 am and 6 pm) except Day 9 when only one injection was applied in the morning. The compounds were administered in a “cocktail” which was prepared daily as follows: first the proper amount of MS-PPOH (prepared at Eötvös Loránd Research Network – Research Centre for Natural Sciences, Budapest, Hungary) was dissolved in 30% hydroxy-propyl-β-cyclodextrin (HPβCD, Cyclolab Ltd., Budapest, Hungary) in an ultrasonic water-bath (40 min, 24 °C). Then indomethacin (Sigma-Aldrich Co., St. Louis, USA) was added and sonicated for further 50 min. Last, L-NAME (Merck KGaA, Darmstadt, Germany) was added to the solution which dissolved immediately. Nine animals were treated with this cocktail, while 8 control rats received 30% HPβCD solution. The dosing volume was 2 mL/kg.

#### Treatment allocation

Based on individual numbering, the first two animals served as controls and the next two as treated across two cages, while in two additional cages this allocation was reversed. The last animal (in the cage with five) was assigned to the treated group. This constituted a balanced allocation rather than true randomization, resulting in nearly identical baseline body weights between groups. Separate persons performed the injections and the measurements; those who did the latter were not aware of which treatment the animals received.

**Fig. 1:**
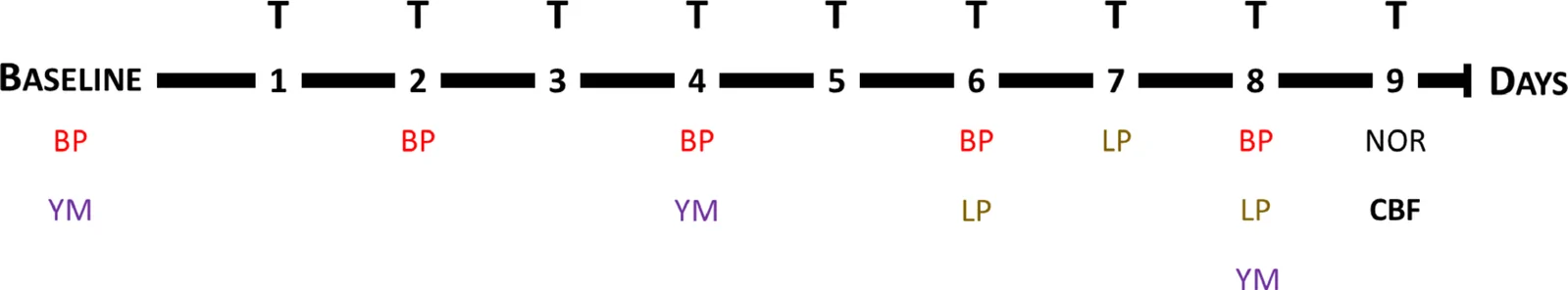
Timeline of Exp 1. All interventions are indicated by corresponding abbreviations on days when they were performed. BP: blood pressure measurement, YM: Y-maze alternation test, LP: lever press learning, NOR: novel object recognition assay, CBF: cerebral blood flow measurement, T: treatment.

The timeline of the experiment is shown in Fig. 1

#### Cognitive assays

##### Y-maze alternation test

The Y-maze used to assess short-term working memory was a symmetrical, dark grey plastic apparatus with three identical arms (45 × 16 × 29.5 cm) arranged at 120-degree angles and connected at the centre. Each arm was accessible through an arched opening in a partition wall. The maze was placed in a room with moderate lighting (20 lx at surface level).

At the beginning of each trial, the rat was placed in the designated “entry” arm (arm #1), and its movements were recorded for 10 min using a ceiling-mounted video camera. The video feed was displayed on a computer monitor (Record-It!, v1.0, Panlab, Harvard Apparatus, Barcelona, Spain) outside the experimental room, where the experimenter manually logged arm entries in real-time in an Excel document. After each trial, the animal was returned to its cage, and the maze was cleaned with 10% alcohol.

The collected data were analysed using an Excel macro to determine the total number of entries and the percentage of spontaneous alternation. Spontaneous alternation was defined as an entry into the least recently visited arm (e.g., 1-2-3-1), with the percentage calculated as:

$$Spontaneous \, alternation \, \% = \left( {\left( {number \, of \, spontaneous \, alternations} \right)/\left( {total \, entries - 2} \right)} \right) \times 100$$

Animals’ performance within the maze was observed at baseline (about a week before the treatment) and on Treatment Day 4 and 8 (*see* Fig. 1).

##### Lever-press training

Animals performed this task from Treatment Day 6 to 8 (*see* Fig. 1) in a 30 × 24 × 21 cm Skinner box (MedAssociates, VT, USA) equipped with a lever and a magazine at the opposite wall of the chamber. During this short period rats had to learn that pressing the lever would result in getting a pellet in the magazine. If the animals pressed the pedal, the feeder light turned on and remained lit until the delivered pellet was eaten. After a 5-s-long intertrial interval the lever was activated again (indicated by a light stimulus above it) until the next lever-press which resulted in the next pellet delivery. The first measurement on Day 6 was 20 min long, the subsequent sessions the next two days lasted for 30 min. The number of rewards obtained during the sessions was the outcome variable.

##### Novel object recognition

This assay was conducted on Treatment Day 9, right after the last injection (*see* Figure S1) as described in Gáspár et al. ^33^. Animal behaviour was observed in a 48 × 48 × 42 cm box lined with bedding material and recorded using a video camera system (Record-It!, v1.0, Panlab, Harvard Apparatus, Barcelona, Spain). The experiment included an acquisition trial and a retention trial. During the acquisition trial, the rats were allowed to explore two identical objects placed in the box for 3 min. Following an 80-min delay, the retention trial was conducted, where one of the original objects was replaced with a different one (novel object), and the rats again had 3 min to explore them. The objects used were a plastic bottle filled with gravel and a glass bottle filled with a blue dye solution. Both objects were equally assigned as familiar or novel. The primary measurement was the exploration time for each object. Recognition memory was assessed using the discrimination index, calculated as:  *DI* = *(t*_*new object*_*—t*_*old object*_*) / (t*_*new object*_ + *t*_*old object*_*).* According to predefined criteria, rats that spent less than 10 s by exploring the objects or examined only one of the two objects during any trial were excluded from the analysis.

#### Blood pressure measurement

The animals’ blood pressure was measured using a 4-channel CODA® apparatus (Non-Invasive Blood Pressure System; Kent Scientific Corp., Torrington, USA), which utilizes two tail cuffs: an occlusion cuff and a volume pressure recording cuff. The animals were placed in holding tubes and the cuffs were put on their tails. The tubes then were placed on a warmed platform and were covered with a black insulating cloth. Each recording session included 40 inflating – releasing cycles of blood pressure measurement. The variables measured were systolic, diastolic, and mean blood pressure, blood flow, and heart rate. Measurement cycles were categorized by the equipment itself as ‘failed’ or ‘OK’ but even from the ‘OK’ set we filtered out any unrealistic data and excluded values that fell outside the mean ± 2 standard deviations. We then calculated a revised mean for each variable and assigned it to the subject as its measured value on the given day.

Each animal underwent a habituation session 7–9 days before the experiment. Prior to the measurements, the transparent restriction tubes were placed inside the animals’ home-cages, in order to help them acclimate being inside of these plastic instruments.

Baseline blood pressure measurement was carried out at 4–6 days before the day of the first treatment. During the treatment, blood pressure was monitored on Days 2, 4, 6 and 8 (*see* Fig. 1).

#### Cerebral blood flow measurement

Collecting data of altered CBF was the same as in our previous publication^9^. On the last day of the experiment, following the NOR test, the animals were anesthetized with intraperitoneal injections of urethane at a dose of 1.5 g/kg prior to surgery. The depth of anaesthesia was verified by the absence of tail-pinch and pedal reflexes. The rats were then positioned in a stereotaxic frame (Stoelting, Wood Dale, IL, USA) and placed on a preheated bench set to 37 °C (Supertech Instruments, Pécs, Hungary). Lidocaine (Egis, Budapest, Hungary) was applied locally on their shaved heads. A midline skin incision was made, and the skull surface was cleaned. The left barrel cortex was located using stereotaxic coordinates (3.3 mm posterior to bregma and 4.5–5.0 mm lateral from the midline ^34^), and an approximately 3 mm wide cranial window was created with a dental drill, avoiding penetration of the dura mater. The window was filled with artificial cerebrospinal fluid (with a standard composition of 126 mM NaCl, 1.8 mM KCl, 1.25 mM KH₂PO₄, 2.4 mM CaCl₂·2H₂O, 26 mM NaHCO₃, 12 mM glucose, and 1.3 mM MgSO₄·7H₂O), and a laser Doppler probe (OxyFlo Probe, Oxford Optronix Ltd. – ADInstruments, PowerLabs) was lowered in the liquid. This probe measured changes in cerebral blood flow in the barrel cortex while the contralateral whisker pad was manually stimulated with a brush at approximately 2 Hz. Data were registered using LabChart 6 software (ADInstruments, Dunedin, New Zealand, version 6.2.1). Changes in perfusion units (PFU) were recorded over time, with a 5 mV difference corresponding to 1 PFU change. The procedure involved positioning the probe, stimulating the whisker pad, and adjusting the probe position if no notable blood flow change was detected. Once a stable signal was obtained, a 2-min baseline was established. The recording protocol included two 30-s contralateral stimulations (see example in Fig. 4b) and one 15-s ipsilateral “control” stimulation, each separated by 3 min of break. This sequence was repeated four times with 3-min intervals between each. The mean of the 15 s long baseline period preceding stimulation was subtracted from the mean of the stimulation period to determine signal amplitude. The amplitudes of the eight contralateral stimulations and the four ipsilateral stimulations (aspecific signals) were averaged. The average ipsilateral amplitude was subtracted from the contralateral amplitude mean, yielding the coupling parameter (increased blood flow) characteristic for each individual.

After the CBF measurement- still under the influence of anaesthesia—animals were swiftly decapitated and prepared for tissue sample harvest.

#### Tissue sampling

Barrel cortices, hippocampi and upper part of small intestine were collected after the CBF assessments. Tissue samples for ELISA tests were washed with phosphate buffered saline (PBS, pH:7.4, in 1:10 dilution), weighed, placed into Eppendorf vials, snap-frozen in liquid nitrogen and stored at −80 °C until further processing. The animals’ small intestines after removal were measured in length. From the proximal end, approximately 10% was discarded and the next 10% was taken as tissue sample. The samples were cut open in longitudinal direction, cleaned of faeces and checked for signs of ulceration or malformation. Lastly, the samples were washed in PBS, then frozen and stored at −80 °C for later evaluation.

##### Epoxyeicosatrienoic acid level determination

For the measurement of 11,12-EET, BlueGene ELISA Kits were used (11,12-Epoxyeicosatrienoic Acid ELISAs kit, BlueGene Biotech, China, Catalogue number: E02E0540). For homogenization, 0.02 mol/L PBS (pH 7.0–7.2) was added to the barrel cortex and hippocampus tissues according to the dilution ratio (*see* homogenization protocols on manufacturer’s website^35^) and crushed by metal ball crushing. If the tissue samples were larger, this step was also preceded by pulverisation in a mortar using liquid nitrogen. The sample was then centrifuged for 15 min. The collected supernatants were stored in a freezer at −80 °C until evaluation. The obtained amount of supernatants of some animal samples were insufficient for testing it in one well of the ELISA kit, so some samples within groups were pooled or left out.

### Exp 2: cognitive effects of the uncoupling cocktail in aged, experienced long-Evans rats

We conducted this study with 25 experienced male Long Evans rats (Janvier Labs, France) weighing 270–490 g and being 27.5 months of age at the start of the experiment. Animals were kept in groups of three (or two if previous cage-mates died). They had restricted food access throughout their life: 45 g for three rats (proportionally varied if needed), with food supplied at least 2 h before the end of the dark phase.

It is important to stress that *experienced* in this context refers to animals that, prior to this experiment, underwent an intensive cognitive training regimen—completing at least one daily cognitive task on weekdays—resulting in over two years of accumulated testing experience. As outlined in the introduction, such subjects provide a translationally relevant model for preclinical studies investigating cognitive dysfunction, as their extensive training history better reflects complex cognitive profiles observed in humans.

**Fig. 2:**
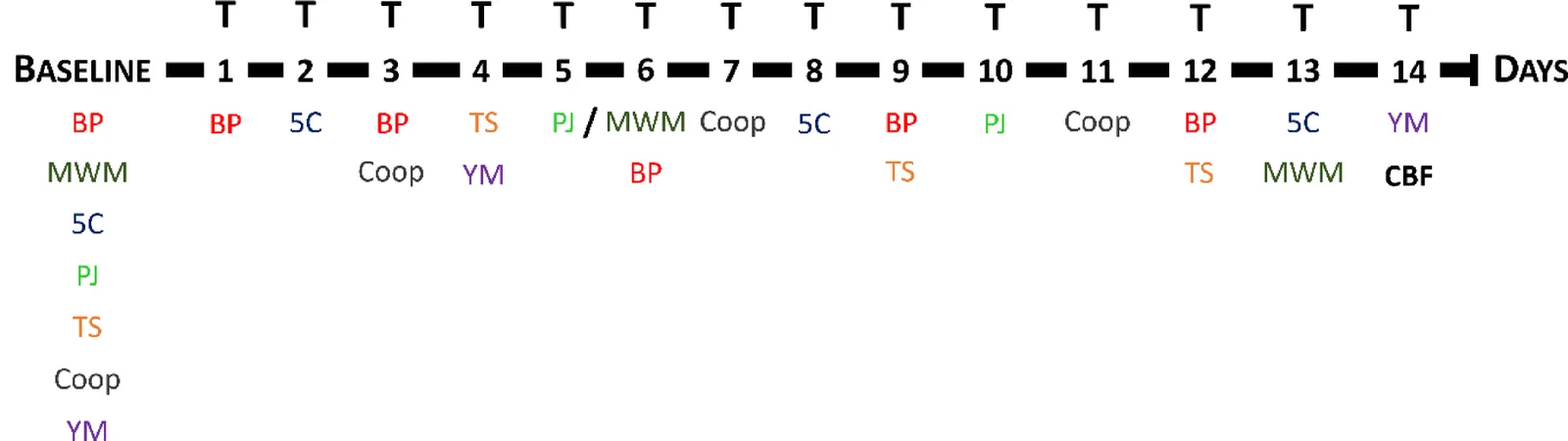
Timeline of Exp 2. All interventions are indicated by corresponding abbreviations on days when they were performed. BP: blood pressure measurement, MWM: Morris water maze, 5C: 5CSRTT, PJ: pot jumping test, TS: touchscreen pairwise visual discrimination, Coop: cooperation task, YM: Y-maze alternation test, CBF: cerebral blood flow measurement, T: treatment. “**/**” indicates that half of the animals did PJ on day 5 and MWM on day 6, while the other half was tested in reverse order.

The timeline of the experiment is shown in Fig. 2.

#### Treatment

For 14 consecutive days (*see* Fig. 1) the animals were treated ip. with the presumed uncoupling cocktail (n = 12) or vehicle (n = 13) twice a day (at 6 am and 6 pm) except on Days 1 and 14 when they only received one injection in the evening and in the morning, respectively. The starting dosages were set to 5 mg/kg for MS-PPOH, 7 mg/kg for L-NAME and 0.7 mg/kg for indomethacin.

The compounds were administered in a “cocktail” which was prepared daily as follows: first the proper amount of MS-PPOH (prepared at Eötvös Loránd Research Network – Research Centre for Natural Sciences, Budapest, Hungary) was dissolved in 45% hydroxy-propyl- β-cyclodextrin (HPβCD, Cyclolab Ltd., Budapest, Hungary) in an ultrasonic water-bath (40 min, 24 °C). Then indomethacin (Sigma-Aldrich Co., St. Louis, USA) was added and sonicated for further 50 min. Last, L-NAME (Merck KGaA, Darmstadt, Germany) was added to the solution which dissolved immediately. Control animals received 45% HPβCD solution. The dosing volume was 2 mL/kg.

The animals were divided into seven cohorts whose treatments and behavioural experiments started with a day shift. In the first treated cohort, after the first four injections we observed that both the vehicle- and cocktail treated animals were sleepy and not motivated to work in the cognitive assays, therefore we reduced the cyclodextrin concentration to 30%. After their tenth injection, because of overt toxicity (*see* Results), we needed to decrease the doses of the components to 2.5 mg/kg MS-PPOH, 5 mg/kg L-NAME and 0.5 mg/kg indomethacin, which were dissolved in 20% HPβCD + 1% NaHCO_3_. The changed solvent resulted in a faster dissolution time through ultrasound sonication (10 min for MS-PPOH and additional 20 min for indomethacin) and a pH = 8.0–8.1 of the “cocktail” solution that was tolerable for the animals, as the pH of the peritoneal fluid is between 7.5 and 8. In case of animals that started to lose weight intensively, drug injections were suspended until they gained weight again. See the detailed treatment and exact number of injections in Supplementary table S1.

#### Treatment allocation

Animals were assigned to vehicle- and drug treated groups in a balanced manner based on their performance in baseline trials (*see* below). Learning parameters from all the assays were taken into account with the help of an in-house made excel macro. Separate persons performed the injections and the learning assays. Those conducting assays were originally unaware of treatment allocation, however, due to toxicity-induced weight loss, blinding could not completely be maintained.

#### Cognitive assays

##### Morris water maze

The apparatus consisted of a black circular pool with a diameter of 190 cm and a depth of 60 cm, filled with water at 40 cm depth and maintained at 23 ± 1 °C. A hidden round black escape platform, 10 cm in diameter, was submerged 0.5 cm below the water surface and located in one of the four quadrants (south-east (SE), south-west (SW), north-east (NE), north-west (NW)), 40 cm from the pool’s edge. Extra-maze cues were positioned on the walls of the experimental room to aid in orientation during the swim trials. At the beginning of each trial, the rat was placed into the pool at one of four starting points (North, East, West, or South, systematically rotated) and given 3 min to find the hidden platform. Each rat was allowed to remain on the platform for 30 s before being removed, dried with a cloth and returned to its home cage. Each animal underwent three trials a day with 20-min intertrial interval between them. Escape latency was recorded, and the swimming path was tracked using Smart v3.0 video tracking system software (Panlab, Barcelona, Spain).

Animals learned the task at their age of 3.5–4 months during 4 daily sessions with the platform fixed in the SE quadrant. Afterwards, they received monthly maintenance training sessions in which the location of the platform was rotated around the four quadrants from session to session. For the current experiment, escape latency values from the last two maintenance sessions preceding the treatment period were averaged for each animal and the obtained mean was considered as baseline performance and used in the group allocation process. During the treatment period MWM task was performed on Day 5/6 and 13 with different platform locations (NW and NE, respectively).

##### Cooperation paradigm

Social learning was assessed in a cooperative task as described in Gáspár et al. ^33^. Pairs of animals participated in the task, which was carried out in a Skinner box measuring 30 × 24 × 21 cm (MedAssociates, VT, USA). The opposite walls of the chamber were equipped with one nose-poke module, one lever and one magazine for each. The two animals were separated by a dividing fence in the midline of the chamber. A trial started with lights turning on in the nose-poke modules for a maximum of 15 s. Any of the animals could initiate the task by continuously nose-poking the module for 3 s, which then activated the lever on the opposite side for 15 s. If the other animal pressed the lever within this period, a reward pellet was delivered for both rats and a new trial started. An omission response was recorded if the rats failed to perform a nose-poke or lever press. Incorrectly timed or out-of-sequence responses resulted in a 5-s timeout. Learning performance was characterized by the percentage of rewarded trials. Each daily test session lasted 20 min.

Animals started to learn cooperation at 6.5–7 months of age. Within 2 months training, most pairs acquired the task, and after that they took part in weekly maintenance sessions. For establishing a baseline for the current experiment learning performance values in the last 3 sessions preceding the treatment period were averaged, and these means were also used for treatment allocation. Cooperation tasks were run on Treatment Day 3, 7 and 11 (*see* Fig. 2).

##### 5-choice serial reaction time task

The 5-choice serial reaction time task (5CSRTT) apparatus featured a test box measuring 31 × 35 × 34 cm (TSE Systems, Bad Homburg vor der Höhe, Germany). Task was based on method described in Gáspár et al. ^33^. The boxes included five nose-poke modules on the back wall and a magazine on the front wall. During the task, following a 5-s inter-trial interval, a 1-s light stimulus appeared in one randomly chosen nose-poke module, prompting the animal to nose-poke into the indicated hole. A response was considered correct if the animal nose-poked into the designated hole during the stimulus or within 5 s afterward (limited hold). Correct responses were rewarded with a pellet delivered into the magazine, and nose-poking into the magazine initiated the next trial. An incorrect response occurred if the animal nose-poked into any hole where the stimulus was absent. An omission was recorded if the rat did not nose-poke within the limited hold period. Incorrect responses and omissions resulted in a 5-s time-out punishment, during which the house light was turned off. After the time-out, the house light was turned back on, and the rat could start the next trial by nose-poking into the magazine. A premature response was recorded if the animal nose-poked into any hole during the inter-trial interval, also resulting in a time-out punishment. Each daily test session lasted 20 min. The measured outcomes included the percentages of omission, correct, and premature responses, and accuracy. Definitions for each were the following formulas:

$$correct \% = (correct \, responses/(omissions + correct \, responses + incorrect \, responses)) \, \times 100$$

$$omission \% = (omissions/(omissions + correct \, responses + incorrect \, responses)) \times 100$$

$$premature \% = \left( {premature \, responses/number \, of \, trials} \right) \times 100$$

$$accuracy \% = ((correct \, responses/(correct \, responses + incorrect \, responses)) \times 100).$$

Animals began to learn 5CSRTT in the first half year of their lives and acquired the skills to perform the abovementioned task in an over 1-month-long training period. Afterwards they performed weekly maintenance tests. For the current experiment, the baseline was determined by averaging data from the last three sessions preceding the treatment. For treatment allocation the correct and premature response ratio was used from these averages. During treatment 5CSRTT was carried out on Day 2, 8 and 13 (*see* Fig. 2).

##### Pot-jumping

The method was based on the study of Ernyey et al.^36^. In the Morris water maze tank, twelve flowerpots (16 cm high and 10 cm wide at the bottom) were positioned upside down to form a circle. The distance between the centres of adjacent pots increased gradually from 18 to 46 cm in a counterclockwise direction. The tank was filled with water to a depth of 6 cm to prevent rats from climbing off the pots. During each session, the animals were placed on the starting pot, which was the closest to the next pot. For three minutes, the rats could move freely on the pots while their behaviour was observed and recorded with a video camera system. The key outcome parameters were the longest distance jumped between pots and the number of passes.

Pot-jumping started around the age of 3.5–4 months. Maintenance sessions were carried out weekly. Baseline was determined by the last two sessions prior to treatment. For treatment allocation two variables, longest distance travelled, and number of passes were used. Pot-jumping was assessed on Day 5/6 and 10 (*see* Fig. 2).

##### Touchscreen—pairwise visual discrimination

The experiment was conducted in a touchscreen apparatus (Campden Instruments Ltd., Lafayette, IN, USA, cat. no. 80604) according to the method in Gáspár et al.^33^. Each box featured a touch screen at the front and a magazine at the back. The touchscreen could be split into two sections with a cover panel. Subjects were trained to distinguish between two images (one correct and one incorrect) that appeared randomly in the left or right window of the touchscreen. Half of the animals got Image 1 as a correct image, for the other half the Image 2 was correct. Selecting the correct image with a nose-poke resulted in a pellet reward, while choosing the incorrect image caused a 5-s timeout during which the house light turned on. The appearance of the two images for the next trial was triggered by the subjects entering and exiting the food magazine. Each daily test session lasted 20 min, and the ABET II touch v2.15 software recorded the number of completed trials as well as correct and incorrect responses.

Similarly to 5CSRTT, animals learned the touchscreen task in the first half year of their lives and acquired the skills to perform the final stage described above in an over 1-month-long training period. Later they performed maintenance tests weekly, as well. For the current experiment, the baseline was derived from averaging data from the last three sessions preceding the treatment. For treatment allocation the correct response ratio was used. After the start of the treatment the touchscreen task was conducted on Day 4, 9 and 12 (*see* Fig. 2).

##### Y-maze alternation test

This test was conducted as it is described in Exp 1*.*

Measurements were carried out three times: between 8 and 11 days before the treatment period (baseline), on Day 4 and Day 14 right after the last injection (*see* Fig. 2).

#### Blood pressure measurement

Blood pressure was assessed as it was described in Exp 1. After 3 habituation measurements within two weeks preceding the treatment period, baseline was recorded on Day 1 prior to the first injection. Additional measurements were carried out on Day 3, 6, 9 and 12 (*see* Fig. 2).

#### Cerebral blood flow measurement

CBF measurements were done in the same manner as described in Exp 1. CBF was measured on Day 14, after the Y-maze test (*see* Fig. 2).

#### Tissue collection and sampling

Brain hemispheres (excluding the cerebellum), upper part of small intestine and both kidneys were collected after the Doppler-probing.

Tissue samples for ELISA tests were prepared and the determination of 11,12-EET levels was done the same way as described in Exp 1.

Samples for histological analysis were put inside tissue cassettes, placed into 4% paraformaldehyde (PFA) then within 48 h into 70% alcohol. Later these were dried and embedded in paraffin wax in preparation for tissue slicing and staining (HistoCore Arcadia C—Cold Plate and HistoCore Arcadia H—Heated Paraffin Embedding Station, Leica, Wetzlar, Germany).

##### Prostaglandin E_2_ (PGE_2_) level determination

The concentrations of PGE_2_ in the small intestine and brain were measured using an ELISA kit from Cayman Chemical (Ann Arbor, MI, USA—Prostaglandin E_2_ ELISA Kit (Monoclonal), Item No. 514010) ^37^. In summary, tissues from the brain and small intestine were homogenized in chilled 100% ethanol with 10 μM indomethacin, followed by centrifugation at 1500×g for 15 min at 4 °C. The ethanol in the supernatants was evaporated using a vacuum centrifuge, and the residues were reconstituted in assay buffer for PGE_2_ measurement. The results were normalized to the tissue weight.

#### Slice preparation and staining

##### Kidney

Samples embedded in paraffin blocks were sliced with a microtome (RM2245 Semi-Automated Microtome, Leica, Wetzlar, Germany), then these sections were stretched onto glass slides (Apex Superior Adhesive Slide) in a water bath (Leica HI1210—Water Bath, Wetzlar, Germany) and finally dried on a heating pad (Leica HI1220—Slide dryer, Leica, Wetzlar, Germany). After complete de-paraffinization and rehydration, staining was done via Harris haematoxylin and counterstaining via eosin. Histological alterations were scored by type and according to their extension in the section. These scores were then summed up for each individual to get a variable for quantifying between group difference.

##### Brain

Six brain samples were randomly chosen from each group for immunostaining. Brains were immersion-fixed in 4% (wt/vol) PFA in 0.1 M(PBS), embedded in paraffin and 6 μm thick tissue sections were cut mounted on pre-coated glass slides (StarFrost). Shortly after deparaffinisation and rehydration, the sections were pre-treated with 0.3% Triton-X 100 for 1 h at 22–24 °C to enhance the penetration of antibodies. Non-specific immunoreactivity was suppressed by incubating our specimens in a cocktail of 5% normal donkey serum (; Jackson, Cambridge, United Kingdom), 2% bovine serum albumin (BSA; Sigma, Darmstadt, Germany) and 0.3% Triton X-100 in PBS for 1 h at 22–24 °C. Sections were exposed (16–72 h at 4 °C) to select combinations of the primary antibodies anti-vesicular glutamate transporter type 1 (VGLUT1, raised in guinea pig, 1:5000, Synaptic Systems, Göttingen, Germany) and anti-glial fibrillary acidic protein (GFAP, raised in rabbit, 1:2000, Synaptic Systems, Göttingen, Germany) diluted in PBS to which 0.1% NDS and 0.3% Triton X-100 had been added. After extensive washing, immunoreactivities were revealed by carbocyanine (Cy) 2 or 3-tagged secondary antibodies raised in donkey (1:200 [Jackson], 2 h at 22–24 °C). Glass-mounted sections were cover-slipped with glycerol/gelatin (GG-1; Sigma, Darmstadt, Germany). Sections were inspected and images acquired on a 780LSM confocal laser-scanning microscope (Zeiss, Jena, Germany) at 20 × primary magnification with emission spectra for each dye were limited as follows: Cy2 (505–530 nm) and Cy3 (560–610 nm).

### Exp 3: Effects of MS-PPOH in young naïve rats

Subjects in this experiment were 16 male Long-Evans rats (Janvier Labs, France, 380–460 g, 6.5-months-old) housed four per cage. Food access was restricted: 60 g/cage supplied at least 2 h before the end of the dark phase.

#### Treatment

In Exp 3, we were interested in the standalone effects of MS-PPOH, thus it was the only compound tested. It was administered ip. for 9 consecutive days, twice daily (at 5 am and 5 pm) except on Days 1 and 9 when only one injection was given in the evening and in the morning, respectively (*see* Figure S2). The dosage was 5 mg/kg, the molecule was dissolved in 20% HPβCD and was administered in 1 ml/kg volume. Odd-numbered animals were allocated to the control groups, even-numbered rats to the MS-PPOH group. This resulted in less than 1% baseline weight difference between groups. Separate persons performed the injections and the assays; those who did the latter were not aware of which treatment the animals received.

#### Y-maze alternation test

**Fig. 3:**
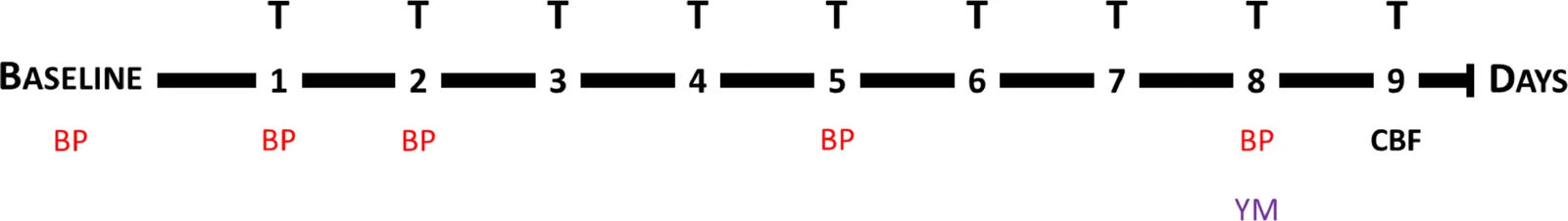
Timeline of Exp 3. All interventions are indicated by corresponding abbreviations on days when they were performed. BP: blood pressure measurement, YM: Y-maze alternation test, CBF: cerebral blood flow measurement, T: treatment.

The test was performed the same way as described in Exp 1. on Treatment Day 8 (*see* Fig. 3).

#### Blood pressure measurement

Blood pressures were measured as described in Exp 1 with an additional refinement measure: cream cheese, also after being introduced to the animals, was applied to the outer rim of the "head-holding" cone. After 3 habituation measurements within a week preceding the treatment period, baseline was recorded on Treatment Day 1 prior to the first injection. Further measurements were carried out on Day 2, 5 and 8 (*see* Fig. 3).

Terminal surgery and CBF measurement was carried out on Day 9 in the same way as described in Exp 1.

#### Tissue collection and sampling

Brain hemispheres (excluding the cerebellum), upper part of small intestine and kidneys were collected after the CBF measurements.

Tissue samples for ELISA tests were preserved the same way as detailed in Exp 1.

ELISA measurements for EET levels and kidney tissue staining (6 samples from each group) were done as detailed in Exp 1 and 2., respectively.

### Exp 4: conditioned taste aversion test with the cocktail in aged rats

To investigate the possible adverse gastrointestinal effect of the treatment cocktail we performed a supplementary conditioned taste aversion (CTA) experiment. In this experiment we used 11 aged male Hannover Wister rats (Toxicoop, Budapest, Hungary) weighing 410–550 g and being 27–27.5 months of age.

For the treatment we used 5 mg/kg MS-PPOH, 1 mg/kg indomethacin and 10 mg/kg L-NAME dissolved in 20% cyclodextrin + 1% NaHCO_3_. Control animals received 20% HPβCD. Animals were treated ip. The experiment spanned six days, during which animals were individually housed and subjected to controlled feeding and fluid intake measurements. Food pellets were provided at 15 g per animal daily, with uneaten food weighed on conditioning and test days (Days 3–6). Animals were randomly allocated to the two treatment groups. Blinding was not feasible because rats required immediate treatment after the first drinking measurement, as the treatment was embedded in the measurement procedure carried out by a single experimenter.

Water deprivation (20 h) began on Day 1, followed by a four hour-long baseline water consumption measurement on Day 2 to establish individual drinking patterns. Conditioning was conducted on Days 3 and 4. Animals were provided with two bottles of 0.3% saccharin solution (Reanal Laboratory Chemicals Ltd., Budapest, Hungary), available for 2 h. On Day 3, after 1 h of saccharin consumption, subjects were treated with either the cocktail or vehicle solution. Thereafter, the saccharin solution was accessible for an additional hour. On Day 4 the same treatment preceded the saccharin access and subsequently, the bottles were provided for two hours. On Days 5–6, animals were provided with a bottle of saccharin solution and a bottle of water for a period of 4 h to evaluate saccharin preference. Solution consumption was monitored by periodic bottle weighing, and residual food weight was recorded. Saccharin preference was determined as the following:

$$saccharin \, preference = saccharin \, solution \, consumption/\left( {saccharin \, solution + water \, consumption} \right) \times 100$$

To validate the CTA method in our hands, we previously carried out a CTA test using the standard positive control, lithium-chloride. The methodical description and results of this experiment are shown in Supplementary Materials.

### Statistical evaluation

In the NVU experiment(s) results obtained with variables that were repeatedly measured (cognitive performance, blood pressure) were evaluated by repeated measures ANOVA. Post-hoc Duncan test was carried out when either a significant treatment effect or a significant interaction was calculated. Results of assays performed at a single time point (CBF, ELISA, immune staining) were evaluated by unpaired t-test. Mann–Whitney U-test was applied for the histological scores of the kidney tissue staining. Saccharin preference in the CTA test was evaluated by unpaired t-test.

## Results

Exp 1: *Dose finding in young naïve Wistar rats* There was no significant difference between groups in any of the measured outcome variables: change of CBF in the barrel cortex in response to stimulation of the contralateral whisker pad, blood pressure, EET levels in hippocampus and barrel-cortex, performance in the Y-maze test. Therefore, the results are not shown here, they can be found in Supplementary materials. Outcome of the lever press and NOR assays was not evaluable due to lack of valid data.

### Exp2: Cognitive effects of the uncoupling cocktail in aged, experienced Long-Evans rats

**Fig. 4:**
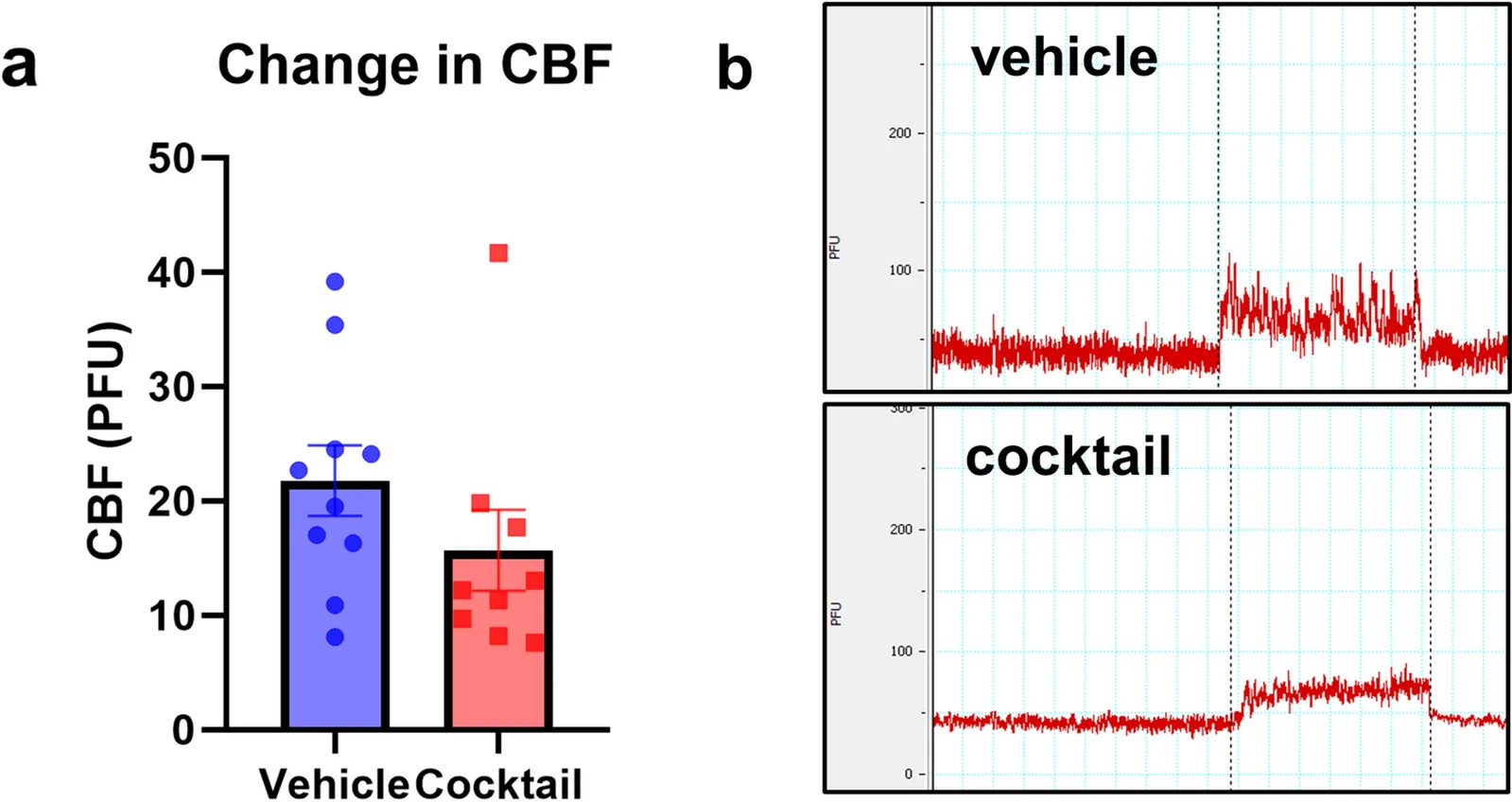
Results of the neurovascular coupling measurement in Exp 2. (**a**) increase in CBF in the barrel cortex in response to whisker stimulation (mean ± SEM) (**b**) exemplary measurement recordings, showing change of CBF (PFU) during a stimulation period. The vertical dotted lines mark the start and end of such periods, outlining 30 s of recordings; ‘vehicle’: vehicle-treated animal’s recording, ‘cocktail’: cocktail-treated animal’s recording.

The uncoupling cocktail only caused a non-significant, 28% decrease in the hyperaemia induced by whisker stimulation and measured in the barrel cortex (Fig. 4a). In three vehicle-treated and 3 cocktail-treated animals we could not detect measurable signals in response to whisker stimulation; thus, they dropped out from this measurement.

**Fig. 5:**
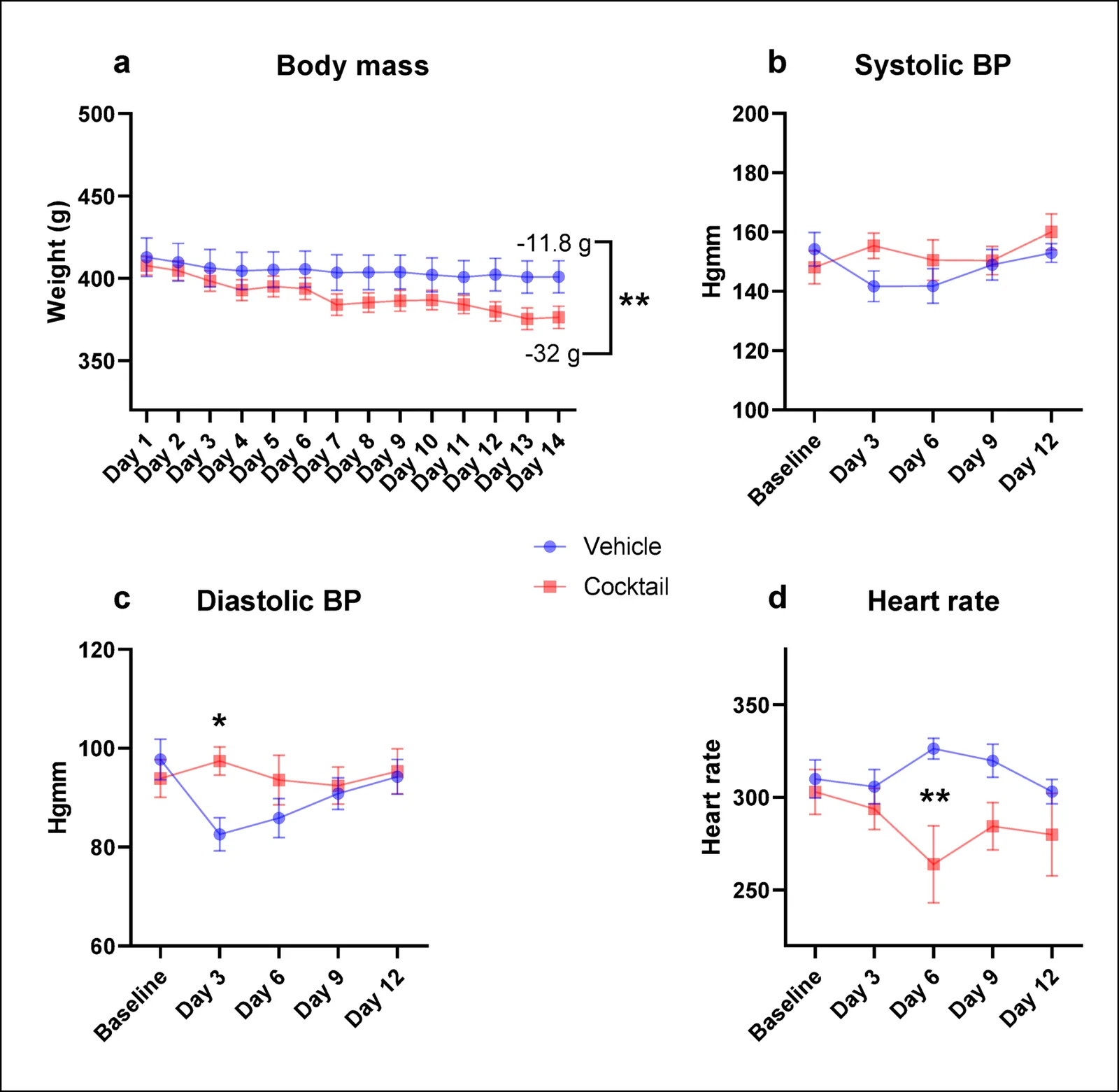
Body mass and blood pressure results in Exp 2. (**a**) Change in body mass of the animals during the treatment period (mean ± SEM). **: *p* < 0.01 vs ‘vehicle’, t-test. (**b**), (**c**) and (**d**) systolic, diastolic blood pressure and heart rate, respectively, over the course of vehicle or cocktail treatment (mean ± SEM). * and **: *p* < 0.05 and *p* < 0.01.

The most conspicuous effect of the treatment was the decreased food intake and the consequent weight loss in the cocktail-treated group (Fig. 5a). As we strived to take all the cocktail treated animals over the course of the experiment this outcome necessitated to introduce breaks in the series of injections at certain timepoints to avoid too much decrease in the body mass and let the animals to recover weight. As a result, animals in the cocktail treated group received varying cumulative doses of the components: between 50 and 75 mg/kg of MS-PPOH, 100 to 138 mg/kg of L-NAME and between 10 and 13.8 mg/kg of indomethacin. Table S1 shows the exact number of cocktail injections and the cumulative dose of the cocktail components which each rat received. Even with these precautionary measures the decrease in body mass by the end of treatment was 7.8% (32 g) in the cocktail treated group while it was only 2.8% (12 g) in the vehicle treated group; the difference was significant (t-test, t(23) = −3.066, *p* < 0.01).

There were not consistent changes in the blood pressure and heart rate of the animals (Fig. 5b–d). No significant effects were detected in systolic blood pressure, while a significant Time x Treatment interaction was found for diastolic blood pressure (F(4, 92) = 2.912, *p* < 0.05), due to a substantially lower value in the vehicle-treated group on Day 3. ANOVA showed a significant Treatment effect in heart rate measurements (F(1, 23) = 2.912, *p* < 0.01); however post hoc analysis only revealed significant difference between the two groups on Day 6.

**Fig. 6:**
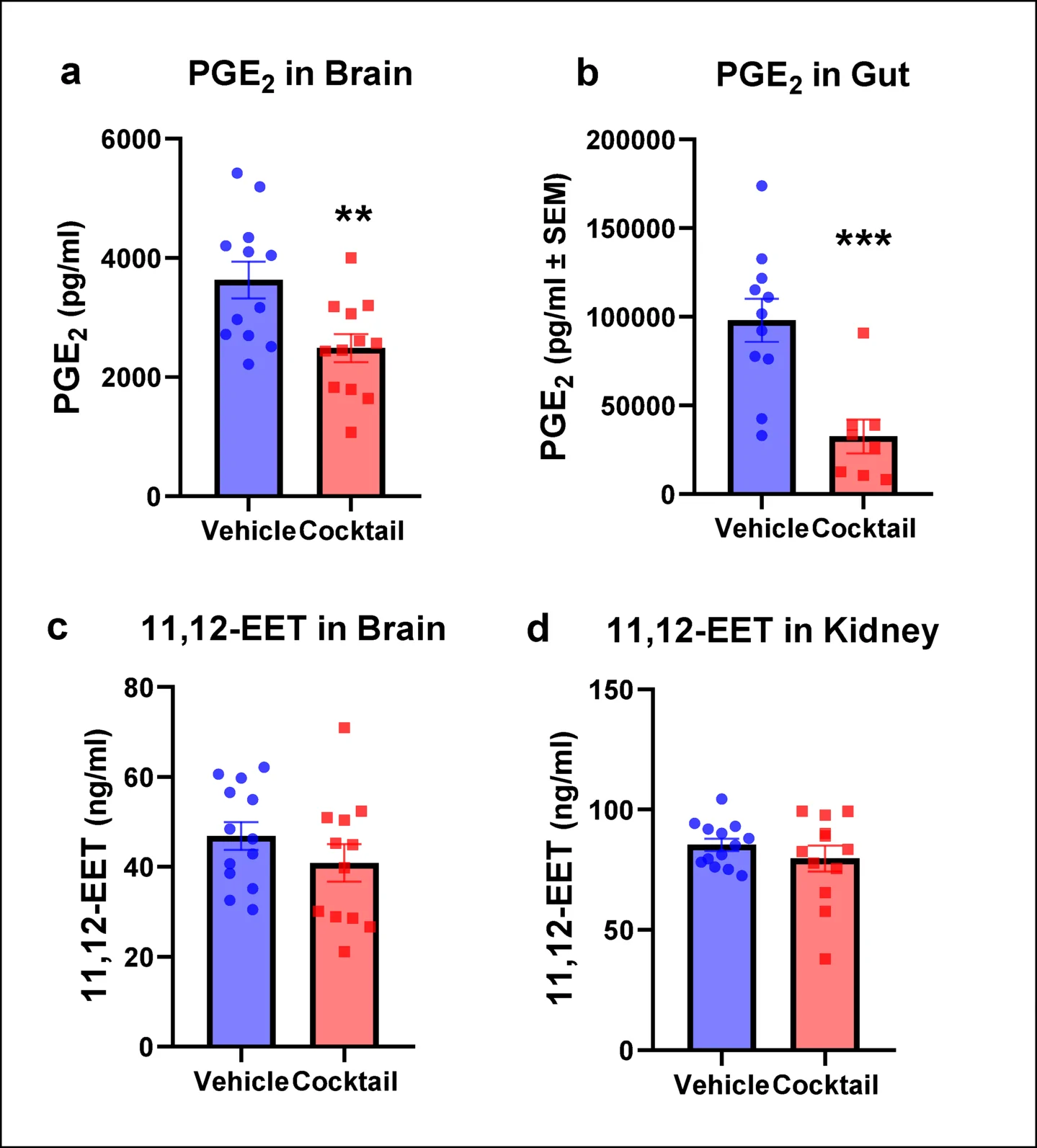
ELISA measurements of PGE_2_ and 11,12-EET in Exp 2. (**a**) and (**b**) PGE_2_ levels in the brain and small intestine, respectively (mean ± SEM). (**c**) and (**d**) 11,12-EET levels in the brain and kidney, respectively (mean ± SEM). ** and ***: *p* < 0.01 and *p* < 0.001, respectively, vs ‘vehicle’, t-test.

PGE_2_ levels were significantly lower in the ‘Cocktail’ group both in the brain (t(22) = −2.954, *p* < 0.01) and in the small intestine (t(17) = −3.991, *p* < 0.001) (Fig. 6a, b). On the other hand, no significant alteration was caused by treatment in the case of 11,12-EET levels, in neither sample type (Fig. 6c, d).

**Fig. 7:**
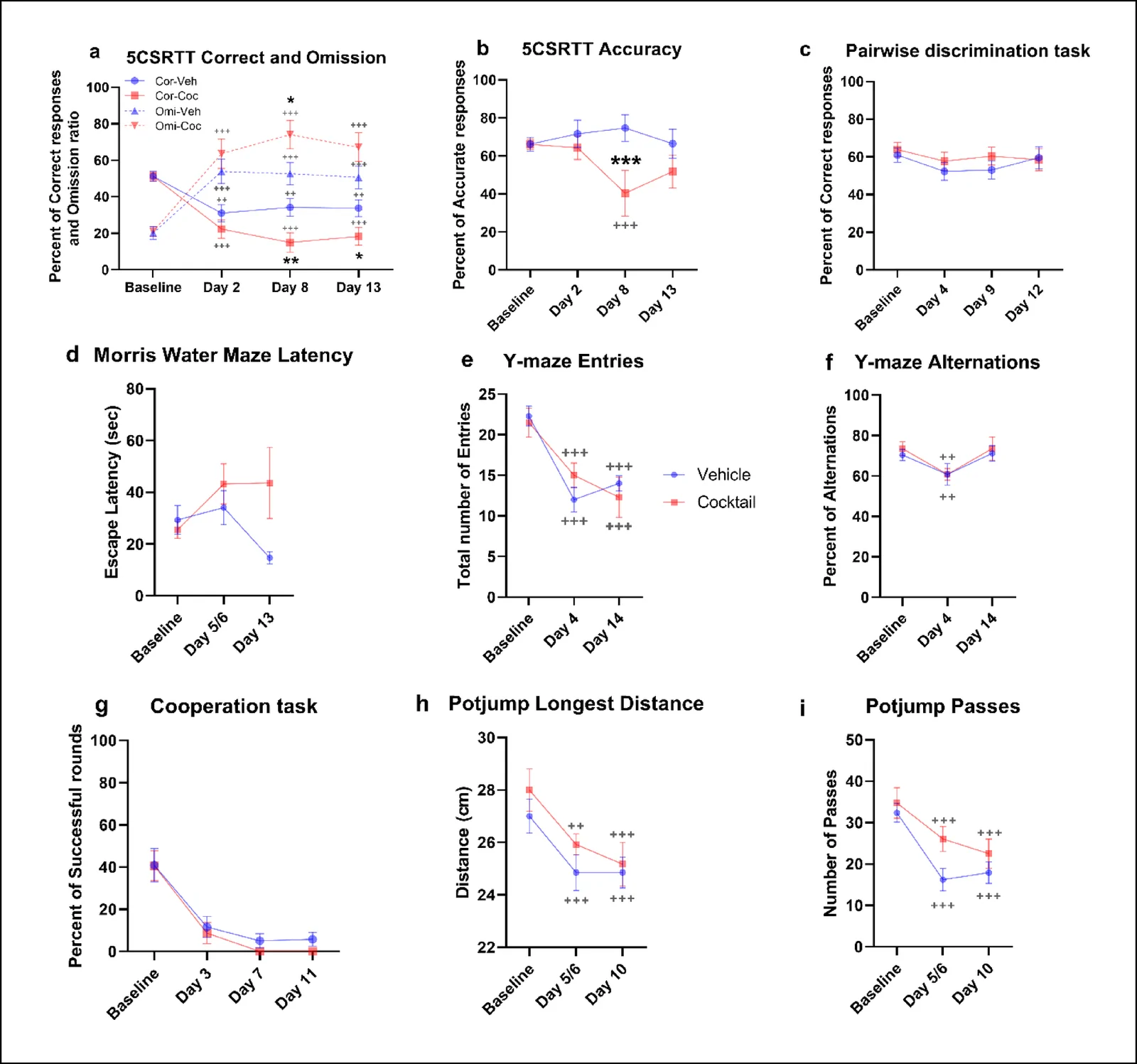
Results in the cognitive tasks in Exp 2 over the course of vehicle or cocktail treatment. (**a**) Correct response percentage and omission percentage in the 5CSRTT. (**b**) Accuracy ratios in the 5CSRTT. (**c**) Correct response rate in the pairwise discrimination task. (**d**) Escape latency in the Morris water-maze task. (**e**) and (**f**) Number of entries and percent of alternations, respectively in the Y-maze. (**g**) Percentage of successful trials in the cooperation task. (**h**) and (**i**) Longest distance covered and number of passes in the potjump test. Mean ± SEM values are shown. *, ** and ***: *p* < 0.05, *p* < 0.01 and *p* < 0.001, respectively, vs ‘vehicle’; + , +  + and +  +  + : *p* < 0.05, *p* < 0.01 and *p* < 0.001, respectively, vs ‘Baseline’; post-hoc Duncan test after repeated measures ANOVA.

Among the cognitive tasks, significant group differences were only observed in the 5CSRTT measurements (Fig. 7a, b). Significant Treatment effects were detected in the percent correct responses (F(1, 23) = 6.947, *p* < 0.05), percent omission responses (F(1, 23) = 4.2524, *p* = 0.0507) and accuracy ratio (F(1, 23) = 7.242, *p* < 0.05). In addition, a significant Time effect occurred in the percent correct and omission responses (F(3,69) = 18.902, *p* < 0.001 and F(3,69) = 23.286, *p* < 0.001, respectively), and a significant Time × Treatment interaction in the accuracy ratio (F(3,69) = 4.149, *p* < 0.01). Correct response rate and accuracy ratio were significantly lower, whereas omission rate was higher in the cocktail-treated group on Day 8.

In MWM, cocktail-treated rats showed a notably higher latency on Day 3 than vehicle-treated rats, but ANOVA did not reveal significant treatment effect or interaction (Fig. 7d). In the other variables, such as percentage of correct responses in the pairwise discrimination task (Fig. 7c), total arm entries (Fig. 7e) and spontaneous alternation percentage (Fig. 7f) in the Y-maze, percentage of successful trials in the cooperation task (Fig. 7g), and longest distance (Fig. 7h) and number of jumps (Fig. 7i) in the pot-jumping test, no notable group difference could be observed.

However, in all assays a decrease in performance from baseline was observed at the first measurement occasion of the treatment period in both groups. This decrease was significant in the 5CSRTT correct % and omission % responses (Fig. 7a), Y-maze arm entries (time effect F(2,46) = 55.215, *p* < 0.001; Fig. 7e) and spontaneous alternations (time effect F(2,46) = 5.298, *p* < 0.01; Fig. 7f), and pot jump longest distance (time effect F(2,46) = 25.291, *p* < 0.001; Fig. 7h) and number of jumps (time effect F(2,46) = 27.683, *p* < 0.001; Fig. 7i) variables.

#### Histology

##### Brain

**Fig. 8:**
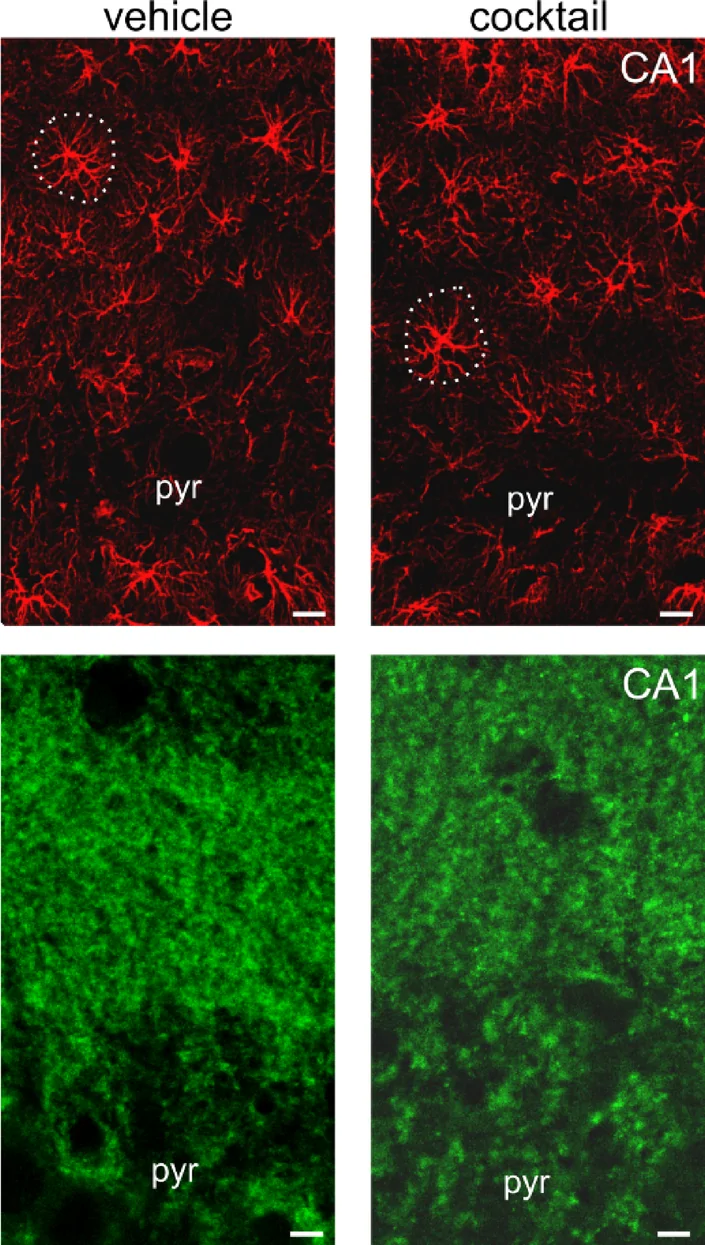
Glial fibrillary acidic protein (GFAP)-immunoreactive astrocytes (red) and vesicular glutamate transporter type 1 (VGLUT1)-immunoreactive terminals in the cornus ammonis 1 (CA1) region of the hippocampus. Dashed lines in top images surround the fields which are covered by the processes of individual astrocytes. Abbreviations: CA1 cornu ammonis 1 region, pyr = pyramidal layer. Scale bar: 10 μm.

We investigated the hippocampus, a vulnerable archicortical brain territory to survey major morphological damage (Fig. 8).

Treatment with the cocktail did not change the density of GFAP^+^ astrocytes in the CA1 region (663.34 ± 52.04 [cocktail] vs. 599.91 ± 51.59 [vehicle], number of astrocytes / 1 mm^2^, *p* = 0.42). The area covered by the processes of individual GFAP^+^ astrocytes were similar in both groups (863.79 ± 46.91 [cocktail] vs. 853.27 ± 43.78 [vehicle], µm^2^, *p* = 0.86). Treatment caused no changes in VGLUT1 fluorescent intensity in the stratum radiatum of the CA1 region (60.44 ± 4.31 [cocktail] vs. 67.19 ± 3.86 [vehicle], arbitrary units, *p* = 0.25).

##### Kidney

**Fig. 9:**
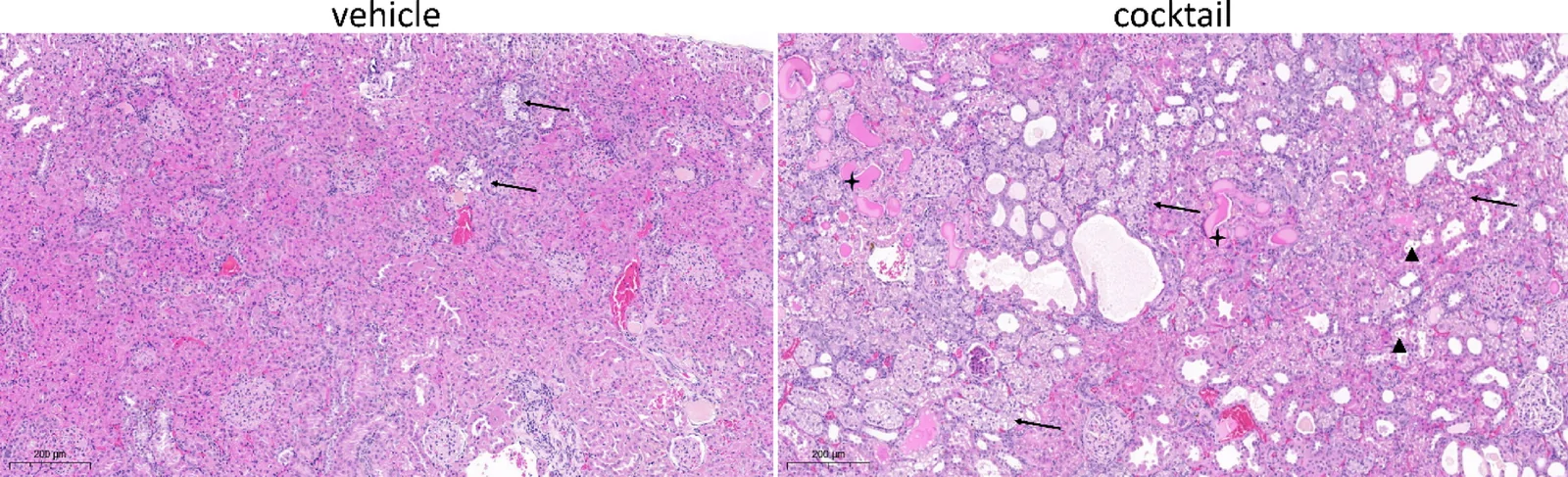
Example sections of kidney histology (haematoxylin—eosin staining). ’Vehicle’: section from a vehicle-treated rat (#A). Focal vacuolation of tubular epithelium (arrow). ’Cocktail’: section from a cocktail-treated rat (#13). Multifocal vacuolation of tubular epithelium (arrow). Multifocal tubular dilation and eosinophilic casts in the tubular lumen (asterisk). Apoptotic cells in the tubules (arrowhead).

Kidney sections from the cocktail-treated group exhibited marked histological alterations (*see* Fig. 9.) compared to vehicle-treated controls (mean histology scores: 10.5 ± 0.96 vs. 1.7 ± 0.36, respectively; *p* < 0.001, Mann–Whitney U-test). For the scoring and categorizations of specific types of renal alterations, see supplementary table S2.

### Exp 3: Effects of MS-PPOH in young naïve rats

MS-PPOH treatment did not have significant effect on whisker stimulation-induced change in CBF, brain EET levels, blood pressure and Y-maze performance. Data can be seen in Supplementary materials.

### Exp 4: Conditioned taste aversion test with the cocktail in aged rats

**Fig. 10:**
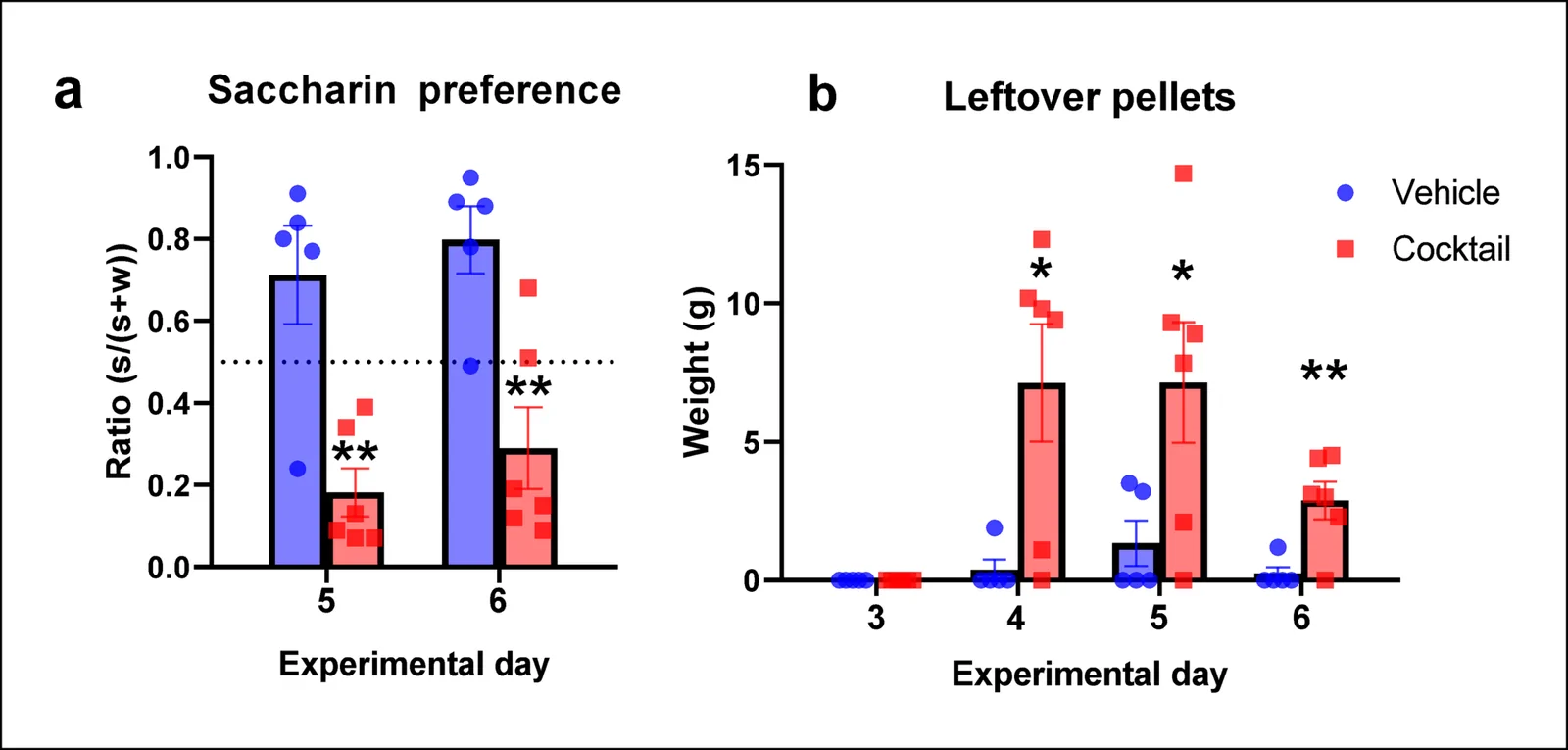
Saccharin consumption and leftover pellets in Exp 4 (**a**) Proportion of saccharin solution consumption in a two-bottle choice situation (saccharin solution and water) after pairing saccharin consumption with cocktail or vehicle treatment (mean ± SEM). The dotted line marks the ‘no-preference’ value. (**b**) weight of leftover food pellets from Day 3 to 6 of their treatment (mean ± SEM). * and **: *p* < 0.05 and *p* < 0.01, respectively, vs ‘Vehicle’ t-test.

Following conditioning, during Days 5–6 when animals had simultaneous access to water and saccharin solution, treated animals demonstrated significantly reduced saccharin preference compared to controls (Day 5: t(9) = 4.219, *p* < 0.01; Day 6: t(9) = 3.813, *p* < 0.01; Fig. 10a).

Additionally, the ‘Cocktail’ treatment group showed significantly greater food pellet leftovers versus controls across multiple days (Day 4: t(9) = −2.842, *p* < 0.05; Day 5: t(9) = −2.306, *p* < 0.05; Day 6: t(9) = −3.406, *p* < 0.01; Fig. 10b).

## Discussion

Our major goal has been to achieve pharmacologically induced NVU in rats, which can serve as a model of human cognitive deterioration. Rats are better and quicker learners than mice, more suitable to study various, more complex cognitive functions. A rat NVU model thus could allow extensive, but at the same time, more specific investigations of the effect of NVU on various cognitive domains.

In Exp 1, halving the doses of MS-PPOH, L-NAME, and indomethacin eliminated the previously observed toxic effects, including hypertension and small intestine ulceration. However, this reduction also abolished the intended NVU, as no significant effects were detected in NVC and working memory. Additionally, brain EET levels remained unchanged.

In our conceptual approach, learnt, experienced animals should be the subjects of studies with cognitive enhancers, as they better reflect the human situation than naïve animals. Concerning pharmacologically induced NVU, they have a particular advantage: the applicable short term (1–2 weeks) drug treatment may not be enough to teach more complex, thus more vulnerable to impairment tasks to the animals, but it may allow to see/follow eventual disruption of already acquired knowledge. In the context of neurodegenerative diseases or age-associated memory impairment, aged rats should naturally be the optimal subjects of these studies.

So, in Exp 2 we turned to an available, aged and cognitively experienced Long-Evans cohort. We employed a modified dosing regimen — higher than in Exp 1 but lower than in our initial study^9^. However, this regimen induced severe toxicity, manifesting as drastically reduced food intake and consequent weight loss. It was certainly an unexpected adverse effect, not seen with younger animals before, which put the whole experiment at stake. Thus, continuous dosing adjustment was necessary to conduct the experiment until the designed end – with the aim to see if the treatment could eventually cause NVU and/or cognitive deterioration at all.

As regards the cognitive capabilities, significant difference between the vehicle- and cocktail-treated groups was only observed in the 5CSRTT. The decreased percentage of correct responses was the direct consequence of the increased percentage of omissions, which may be attributed to the treatment-induced gastrointestinal malaise (decreased motivation for food reward). In contrast, the diminished accuracy on Day 8 appears to be a genuine attentional deficit, as the animals went for the rewards just made incorrect choices. However, the effect was transient as it passed away by Day 13. Poorer performance of the cocktail-treated rats was also observed in the MWM task on Day 13, but it was not statistically significant and was rather a lack of improvement than a real impairment when compared to the vehicle treated group.

In accordance with these rather subtle cognitive impairments, we could only induce mild disruption in neurovascular coupling. Noteworthy, in this aged population the whisker stimulation-induced hyperaemia was quite low even in the vehicle-treated rats, a phenomenon well-known in the literature^6,16,38,39^. In six animals we could not even find appropriate signals. Nevertheless, we deem the obtained 28% inhibition to be biologically meaningful as it falls within the range of significant NVU effects reported in the literature on various kinds of interventions. For example, angiotensin II caused 23% inhibition in mice^40^, while diminishing the number of pericytes (also in mice) resulted in 27% change^15^. The observed significant attention deficit also supports this view. The lack of statistical significance may be attributed to the eventually underpowered state of the NVC measurement. Initially, we started the whole study with 14–14 old, experienced rats, which—according to the a priori power calculations – would have allowed to detect an effect size of ~ 0.8 as significant with a power of 0.66, values similar to those obtained in our previous study^9^. Unfortunately, losing 3 rats due to poor health during the baseline measurements and on the second treatment day, and six others because of the signal detection difficulties resulted in effective sample sizes of 9 and 10, too low to achieve statistical significance for the obtained effect size of 0.6.

Adverse blood pressure effects were not observed. ELISA analyses revealed a moderate decrease in brain PGE_2_, reflecting partial cyclooxygenase inhibition, an effect attributed to indomethacin. No change was observed in brain EET levels, which we used as a biomarker of MS-PPOH effect. Exp 3 yielded similar negative results. Unfortunately, no comparable data are reported in the literature. (The effect of the compounds on brain EET and PGE_2_ levels was not reported in the mouse study^32^ either.) One may speculate about the possible poor brain penetration of MS-PPOH, but no information on it can be found in the literature. However, 11,12-EET was also not decreased in the kidney in either of our experiments. Renal EET levels after intravenous (iv.) administration of MS-PPOH are available from rats in publications. The most frequent dosage used is 20 mg/kg/day, which caused from 28 to 100% reduction after 3–4 days administration depending on experimental subjects (pregnant, salt resistant and cirrhotic rats) ^41–43^, but in rabbits a 2 mg/kg iv. dose followed by 1.5 mg/kg/h infusion for 45 min could produce 38% inhibition. In cardiac tissue a single 3 mg/kg iv. dose was able to decrease the EET by 40–55% in rats^44^. As our dosing parameters (10 mg/kg/day ip. for 13 (Exp 2) or 8 days (Exp 3)) differ in all aspects (route, dose, duration of treatment, subjects) from the above, a fair comparison of drug exposures can hardly be made.

No histological alteration was detected in the brain, while moderate toxicity was noted in the kidney sections. Nephrotoxic effects of all the three compounds have been published in the literature, though typically at higher doses than those applied in the cocktail^45–50^. In Exp 3 in the current study, MS-PPOH did not cause notable histological alterations in kidney samples. However, all these data were obtained in young animals; aged subjects may be more sensitive to the effects of these drugs.

To investigate the cause of reduced food intake in Exp 2, in Exp 4 we performed a CTA paradigm with the cocktail. As the effect was only observed in aged animals, we wanted to run the CTA measurement in aged rats, too, for which purpose Wistar rats were available. The results confirmed that the drug cocktail induces gastrointestinal malaise, explaining the observed feeding disruptions and weight loss.

This finding underscores a critical limitation of the current study: it is conceivable that gastrointestinal malaise and the consequent physiological stress themselves (or other, less overt side effects) may have affected the measured behavioural and physiological variables independently of an impairment in the neurovascular unit. Furthermore, it cannot be excluded that the observed mild NVU may also have resulted from these indirect effects as several pathological conditions (hypertension, diabetes, hypoxia, even acute restraint stress) are known to reduce the efficiency of neurovascular coupling^14,20,40,51–53^.

Another question worth discussing is whether species differences may underlie the failed translation of the model from mice to rats. Non-pharmacological interventions such as restraint stress or ageing induced similar changes in reactive hyperaemia in both mice^5,51^ and rats^38,54^. So, the assumed species-specific difference may lie in the pharmacological sensitivity to the drugs. In our case, the doses of the compounds applied in mice are knowingly toxic in rats. There are differences in the expression levels and inducibility of the target enzymes of the compounds applied^55–58^. Mice are more prone to switch from Cox-1 to Cox-2 in response to inflammatory stimuli^59^ and for endothelial NO synthase uncoupling^60^, which may contribute to neurovascular uncoupling in inflammatory and oxidative stress^20^. The epoxygenase enzyme system has more isoenzymes in mice^61,62^, and its EET product distribution is more diverse^56,62^.

## Conclusions (lessons and challenges)

The development of a reliable pharmacologically induced neurovascular uncoupling model holds significant potential not only for advancing our understanding of cerebrovascular contributions to cognitive impairment but also as a translationally relevant and valid tool for investigating putative cognitive enhancer compounds in the course of drug discovery. However, our findings so far have demonstrated that the current methodological approach — based on a triple enzyme inhibition (EET synthesis, NO production, and prostaglandin inhibition) — faces critical limitations that hinder its suitability as a robust NVU model.

1. Ideally, before jointly administering the compounds, individual dose–response curves should be taken first by measuring EET, PGE_2_ and NO levels to establish a dose that induces sufficient decrease in the brain level of the corresponding enzyme product. (*N.B.*, this kind of checking was not carried out in the mouse study, either^32^). Without it, we do not know what the contributions of the components to the overall NVU effect are. For example, EET results of Exp 2 and Exp 3 show that MS-PPOH may have been inactive at the applied doses. In case of this compound a further uncertainty is that its brain penetration is not known.
2. A bigger challenge lies in finding doses with sufficient cerebral effect but without marked peripheral effects. An important lesson of our experiments is that dose reduction alone proved insufficient to balance efficacy and toxicity, as adjusted doses either failed to induce NVU (Exp 1) or caused severe side effects (Exp 2). Pronounced peripheral toxicity may outweigh the intended NVU disruption and confound the interpretation of cognitive and physiological outcomes.
3. The above problem, in principle, could be solved by direct intracerebral administration of the compounds, optimally via osmotic minipumps connected to an intracerebroventricular (icv.) canula. An additional ‘pro’ for the icv. administration is it would cut through the problem of the unknown brain penetration of MS-PPOH. (Intracisternally administered MS-PPOH blocked basal forebrain stimulation-induced CBF increase in rats^63^.) Assuming that the minipump formulation can be solved, the effective-dose titration experiments mentioned under point 1 should be done using the icv. route.
4. Even with the established individual effective doses, synergistic interaction among the compounds may be expected (see for example^48,50^), which may require further complementary measurements of EET, PGE_2_ and NO levels after the joint administration. This interaction may also explain why even reduced doses in aged animals triggered severe side effects, whereas younger rats tolerated higher doses with fewer complications.
5. Having overcome all the dosing issues, the next challenge relates to the measurement of neurovascular coupling itself. Another lesson from our experiments was that the measurable CBF signals in response to whisker stimulation in the barrel cortex varied from subject to subject, that is, a smaller signal may not reflect disrupted coupling. Because of this variability, the uncoupling efficacy of any intervention should be measured either in much larger samples or – and that would be the optimal – in a self-controlled way (‘before-after’ design). Obviously, it would require a chronic survival model, applying a much less or completely non-invasive measuring method, such as fMRI or fUS. Such a model would also allow longitudinal follow-up of the progression/duration of the induced NVU.
6. Although measuring whisker stimulation-induced hyperaemia in the barrel cortex is very practical, in relation to cognitive functions it is not the most appropriate brain region. Measuring NVC in more relevant subcortical structures (e.g. in the hippocampus) yet again require a more sophisticated and challenging methodology. Standardizing the circumstances of CBF recording (location, timing, stimulus parameters) across studies presents a further challenge.
7. Finally, in our former studies we observed that experienced animals showed some resilience to cognitive impairments^29,30,33^. The transient character of the attention deficit in the 5CSRTT in Exp 2 suggests It may happen in case of NVU, too. As an alternative or complementary explanation, it may also be conceived that cellular and molecular mechanisms (e.g. increased enzyme synthesis) may counteract/compensate the exogenous enzyme inhibition in the long term.

Model development should be a crucial part of pharmaceutical R&D for ensuring a seamless translation from preclinical findings to clinical efficacy. Although the published mouse study^32^ still lacked some additional validation steps (checking the direct enzyme inhibitory effect of the compounds, examining the duration and (ir)reversibility of the produced neurovascular uncoupling and cognitive effects), transferring the pharmacologically-induced NVU model to rats held the promise of providing a relatively simple, attractive method, utilizable for dementia target validation or even for drug screening. The lessons from our experiments highlight that achieving this aim is far more technology-, resource- and time-demanding with questionable feasibility. Nevertheless, these translational difficulties must be communicated, because developmental decisions based on results obtained in not properly validated models are a major source of late-stage clinical failures.

## Supplementary Information

Below is the link to the electronic supplementary material.

Supplementary Material 1

## Data Availability

The datasets used and/or analysed during the current study are available from the corresponding author on reasonable request.

## Abbreviations

5C/5CSRTT: 5-Choice serial reaction time task
AD: Alzheimer’s disease
BBB: Blood–brain barrier
BP: Blood pressure
BSA: Bovine serum albumin
CA1: Cornus ammonis 1
CBF: Cerebral blood flow
Coop: Cooperation
COX: Cyclooxygenase
CTA: Conditioned taste aversion
Cy: Carbocyanine
EET: Epoxyeicosatrienoic acid
Exp: Experiment
GFAP: Glial fibrillary acidic protein
GG: Glycerol/gelatin
HPβCD: Hydroxypropyl-β-cyclodextrin
icv.: Intracerebroventricular(ly)
ip.: Intraperitoneal(ly)
iv.: Intravenous
L-NAME: L-NG-nitroarginine methyl ester
LP: Lever press
MS-PPOH: N-(methylsulfonyl)-2-(2-propynyloxy)-benzenehexanamide
MWM: Morris water maze
NDS: Normal donkey serum
NE: North-east
NO: Nitric oxide
NVC: Neurovascular coupling
NVU: Neurovascular uncoupling
NW: North-west
PBS: Phosphate buffered saline
PFA: Paraformaldehyde
PFU: Perfusion unit
PGE_2_: Prostaglandin E_2_
PJ: Pot jumping
SE: South-east
SW: South-west
T: Treatment
TS: Touchscreen
VGLUT1: Vesicular glutamate transporter type 1
YM: Y-maze
