# Supplementary material for: Translational difficulties in establishing a pharmacologically induced neurovascular uncoupling model in rats
Source: Sci Rep. 2026 Jun 17;16:27671. doi: 10.1038/s41598-026-58244-0 (PMC13542137; doi:10.1038/s41598-026-58244-0)
Supplement: Supplementary file 1 [file 41598_2026_58244_MOESM1_ESM.docx]

*Translational difficulties in establishing a pharmacologically induced neurovascular uncoupling model in rats*

Bence Tamás Varga^1,2^, Aliz Judit Ernyey^1,2^, Brigitta Tekla Tajti^1,2^, Attila Gáspár^1,2^, Zsuzsanna Demeter^1,2^, Levente Kollár^3^, Péter Kovács^3^, Mihály Albert^4^, Alán Alpár^5^, István Gyertyán^1,2,*^

1 - Department of Pharmacology and Pharmacotherapy, Semmelweis University, Budapest, Hungary

2 - Center for Pharmacology and Drug Research & Development, Semmelweis University, Budapest, Hungary

3 - Medicinal Chemistry Research Group and Drug Innovation Centre, HUN-REN Research Centre for Natural Science, Budapest, Hungary

4 - CEVA-Phylaxia Ltd. (Ceva Sante Animale), Budapest, Hungary

5 - Department of Anatomy, Histology and Embryology, Semmelweis University Budapest, Hungary

* Corresponding Author: István Gyertyán

Correspondence to [gyertyan.istvan@semmelweis.hu](mailto:gyertyan.istvan@semmelweis.hu)

Supplementary materials

**Methods:**

**Exp 1***:* *Dose Finding in Young Naïve Wistar Rats*

For this study, we used 17 male Hannover Wistar rats (TOXI-COOP, Budapest, Hungary) weighing 155-215 g and being 2 months of age at the start of the experiment.

Treatment

For inducing NVU the following drugs were used: 2.5 mg/kg MS-PPOH, 0.5 mg/kg indomethacin, 5 mg/kg L-NAME, injected IP for 9 consecutive days, twice per day (at 6 am and 6 pm) except Day 9 when only one injection was applied in the morning. The compounds were administered in a “cocktail” which was prepared daily as follows: first the proper amount of MS-PPOH (prepared at Eötvös Loránd Research Network – Research Centre for Natural Sciences, Budapest, Hungary) was dissolved in 30% hydroxy-propyl- β-cyclodextrin (HPβCD, Cyclolab Ltd., Budapest, Hungary) in an ultrasonic water-bath (40 min, 24°C). Then indomethacin (Sigma-Aldrich Co., St. Louis, USA) was added and sonicated for further 50 min. Last, L-NAME (Merck KGaA, Darmstadt, Germany) was added to the solution which dissolved immediately. Nine animals were treated with this cocktail, while 8 control rats received 30% HPβCD solution. The dosing volume was 2 mL/kg.

The timeline of the experiment is shown in Figure S1

*Fig. S1 – Timeline of Exp 1. All interventions are indicated by corresponding abbreviations on days when they were performed**. BP: blood pressure measurement, YM: Y-maze alternation test, LP: lever press learning, NOR: novel object recognition assay, CBF: cerebral blood flow measurement, T: treatment.*

Cognitive assays:

Y-maze alternation test

The Y-maze used to assess short-term working memory was a symmetrical, dark grey plastic apparatus with three identical arms (45 x 16 x 29.5 cm) arranged at 120-degree angles and connected at the centre. Each arm was accessible through an arched opening in a partition wall. The maze was placed in a room with moderate lighting (20 lx at surface level).

At the beginning of each trial, the rat was placed in the designated “entry” arm (arm #1), and its movements were recorded for 10 minutes using a ceiling-mounted video camera. The video feed was displayed on a computer monitor (Record-It!, v1.0, Panlab, Harvard Apparatus, Barcelona, Spain) outside the experimental room, where the experimenter manually logged arm entries in real-time in an Excel document. After each trial, the animal was returned to its cage, and the maze was cleaned with 10% alcohol.

The collected data were analysed using an Excel macro to determine the total number of entries and the percentage of spontaneous alternation. Spontaneous alternation was defined as an entry into the least recently visited arm (e.g., 1-2-**3-1**), with the percentage calculated as:
*Spontaneous alternation % = ((number of spontaneous alternations) / (total entries - 2)) × 100.*

Animals’ performance within the maze was observed at baseline (about a week before the treatment) and on Treatment Day 4 and 8 (*see* Figure S1).

Lever-press training

Animals performed this task from Treatment Day 6 to 8 (*see* Figure 1) in a 30 × 24 × 21 cm Skinner box (MedAssociates, VT, USA) equipped with a lever and a magazine at the opposite wall of the chamber. During this short period rats had to learn that pressing the lever would result in getting a pellet in the magazine. If the animals pressed the pedal, the feeder light turned on and remained lit until the delivered pellet was eaten. After a 5-seconds-long intertrial interval the lever was activated again (indicated by a light stimulus above it) until the next lever-press which resulted in the next pellet delivery. The first measurement on Day 6 was 20 minutes long, the subsequent sessions the next two days lasted for 30 minutes. The number of rewards obtained during the sessions was the outcome variable.

Novel object recognition

This assay was conducted on Treatment Day 9, right after the last injection (*see* Figure S1) as described in Gáspár et al (36). Animal behaviour was observed in a 48 × 48 × 42 cm box lined with bedding material and recorded using a video camera system (Record-It!, v1.0, Panlab, Harvard Apparatus, Barcelona, Spain). The experiment included an acquisition trial and a retention trial. During the acquisition trial, the rats were allowed to explore two identical objects placed in the box for 3 minutes. Following an 80-minute delay, the retention trial was conducted, where one of the original objects was replaced with a different one (novel object), and the rats again had 3 minutes to explore them. The objects used were a plastic bottle filled with gravel and a glass bottle filled with a blue dye solution. Both objects were equally assigned as familiar or novel. The primary measurement was the exploration time for each object. Recognition memory was assessed using the discrimination index, calculated as *DI = (t_new object_ - t_old object_) / (t_new object_ + t_old object_).* Rats that spent less than 10 seconds by exploring the objects or examined only one of the two objects during any trial were excluded from the analysis.

Blood pressure measurement

The animals' blood pressure was measured using a 4-channel CODA® apparatus (Non-Invasive Blood Pressure System; Kent Scientific Corp., Torrington, USA), which utilizes two tail cuffs: an occlusion cuff and a volume pressure recording cuff. The animals were placed in holding tubes and the cuffs were put on their tails. The tubes then were placed on a warmed platform and were covered with a black insulating cloth. Each recording session included 40 inflating – releasing cycles of blood pressure measurement. The variables measured were systolic, diastolic, and mean blood pressure, blood flow, and heart rate. Measurement cycles were categorized by the equipment itself as ‘failed’ or ‘OK’ but even from the ‘OK’ set we filtered out any unrealistic data and excluded values that fell outside the mean ± 2 standard deviations. We then calculated a revised mean for each variable and assigned it to the subject as its measured value on the given day.

Each animal underwent a habituation session 7-9 days before the experiment. Prior to the measurements, the transparent restriction tubes were placed inside the animals’ home-cages, in order to help them acclimate being inside of these plastic instruments.

Baseline blood pressure measurement was carried out at 4-6 days before the day of the first treatment. During the treatment, blood pressure was monitored on Days 2, 4, 6 and 8 (*see* Figure 1).

Cerebral blood flow measurement

Collecting data of altered CBF was the same as in our previous publication(9). On the last day of the experiment, following the NOR test, the animals were anesthetized with intraperitoneal injections of urethane at a dose of 1.5 g/kg prior to surgery. The depth of anaesthesia was verified by the absence of tail-pinch and pedal reflexes. The rats were then positioned in a stereotaxic frame (Stoelting, Wood Dale, IL, USA) and placed on a preheated bench set to 37°C (Supertech Instruments, Pécs, Hungary). Lidocaine (Egis, Budapest, Hungary) was applied locally on their shaved heads. A midline skin incision was made, and the skull surface was cleaned. The left barrel cortex was located using stereotaxic coordinates (3.3 mm posterior to bregma and 4.5–5.0 mm lateral from the midline (37)), and an approximately 3 mm wide cranial window was created with a dental drill, avoiding penetration of the dura mater. The window was filled with artificial cerebrospinal fluid (with a standard composition of 126 mM NaCl, 1.8 mM KCl, 1.25 mM KH₂PO₄, 2.4 mM CaCl₂·2H₂O, 26 mM NaHCO₃, 12 mM glucose, and 1.3 mM MgSO₄·7H₂O), and a laser Doppler probe (OxyFlo Probe, Oxford Optronix Ltd. – ADInstruments, PowerLabs) was lowered in the liquid. This probe measured changes in cerebral blood flow in the barrel cortex while the contralateral whisker pad was manually stimulated with a brush at approximately 2 Hz. Data were registered using LabChart 6 software (ADInstruments, Dunedin, New Zealand, version 6.2.1). Changes in perfusion units (PFU) were recorded over time, with a 5 mV difference corresponding to 1 PFU change. The procedure involved positioning the probe, stimulating the whisker pad, and adjusting the probe position if no notable blood flow change was detected. Once a stable signal was obtained, a 2-minute baseline was established. The recording protocol included two 30-second contralateral stimulations and one 15-second ipsilateral "control" stimulation, each separated by 3 minutes of break. This sequence was repeated four times with 3-minute intervals between each. The mean of the 15 s long baseline period preceding stimulation was subtracted from the mean of the stimulation period to determine signal amplitude. The amplitudes of the eight contralateral stimulations and the four ipsilateral stimulations (aspecific signals) were averaged. The average ipsilateral amplitude was subtracted from the contralateral amplitude mean, yielding the coupling parameter (increased blood flow) characteristic for each individual.

After the CBF measurement- still under the influence of anaesthesia - animals were swiftly decapitated, and prepared for tissue sample harvest.

Tissue sampling:

Barrel cortices, hippocampi and upper part of small intestine were collected after the CBF assessments.

Tissue samples for ELISA tests were washed with phosphate buffered saline (PBS, pH:7.4, in 1:10 dilution), weighed, placed into Eppendorf vials, snap-frozen in liquid nitrogen and stored at -80°C until further processing. The animals’ small intestines after removal were measured in length. From the proximal end, approximately 10% was discarded and the next 10% was taken as tissue sample. The samples were cut open in longitudinal direction, cleaned of faeces and checked for signs of ulceration or malformation. Lastly, the samples were washed in PBS, then frozen and stored at -80°C for later evaluation.

Epoxyeicosatrienoic Acid level determination

For the measurement of 11,12-EET, BlueGene ELISA Kits were used (11,12-Epoxyeicosatrienoic Acid ELISAs kit, BlueGene Biotech, China, Catalogue number: E02E0540). For homogenization, 0.02 mol/L PBS (pH 7.0-7.2) was added to the barrel cortex and hippocampus tissues according to the dilution ratio (*see* homogenization protocols on manufacturer’s website(39)) and crushed by metal ball crushing. If the tissue samples were larger, this step was also preceded by pulverisation in a mortar using liquid nitrogen. The sample was then centrifuged for 15 minutes. The collected supernatants were stored in a freezer at -80°C until evaluation. The obtained amount of supernatants of some animal samples were insufficient for testing it in one well of the ELISA kit, so some samples within groups were pooled or left out.

**Exp 2:** *Cognitive effects of the uncoupling cocktail in aged, experienced Long-Evans rats*

***Table S1.*** *Number of injections and cumulative doses used for the different cohorts of rats during the treatment period. - In the second column the numbers of rats are shown treated with vehicle or “cocktail”. CD stands for hydroxypropyl-β-cyclodextrin. In the 3^rd^, 4^th^ and 5^th^ column the number of injections of the used vehicle are indicated for the given rats. In the last three columns the cumulative doses of the given substances are shown. The ‘/’ signs in the cells separate the values for different animals.*

| vehicle | | 45% CD | 30% CD | 20% CD  + 1% NaHCO3 | Cumulative dose (mg/kg) | | |
| --- | --- | --- | --- | --- | --- | --- | --- |
| dose of MSPPOH/L-NAME/Indomethacin | | 5-7-0.7 mg/kg | | 2.5-5-0.5 mg/kg | MS-PPOH | L-NAME | Indomethacin |
| Cohort A | 2, vehicle | 4 | 6 | 16 |  |  |  |
|  | 2, cocktail | 4 | 6 | 8 | 70 | 110 | 11 |
| Cohort B | 4, vehicle | 2 | 6 | 18 |  |  |  |
| Cohort C | 2, vehicle |  | 6 | 20 |  |  |  |
|  | 1, cocktail |  | 6 | 12 | 60 | 102 | 10.2 |
| Cohort D | 1, vehicle |  | 4 | 22 |  |  |  |
|  | 2, cocktail |  | 4 | 18/22 | 65/75 | 118/138 | 11.8/13.8 |
| Cohort E | 2, vehicle |  |  | 26 |  |  |  |
|  | 2, cocktail |  |  | 21/26 | 52.5/65 | 105/130 | 10.5/13 |
| Cohort F | 4, cocktail |  |  | 21/24/  26/26 | 52.5/60/  65/65 | 105/120/  130/130 | 10.5/12/  13/13 |
| Cohort G | 2, vehicle |  |  | 26 |  |  |  |
|  | 1, cocktail |  |  | 20 | 50 | 100 | 10 |

**Exp 3:** *Effects of MS-PPOH in young naïve rats*

Treatment

In Exp 3, we were interested in the standalone effects of MS-PPOH, thus it was the only compound tested. It was administered intraperitoneally for 9 consecutive days, twice daily (at 5 am and 5 pm) except on Days 1 and 9 when only one injection was given in the evening and in the morning, respectively (*see* Figure S2). The dosage was 5 mg/kg, the molecule was dissolved in 20% HPβCD and was administered in 1 ml/kg volume.

*Fig. S2 – Timeline of Exp 3. All interventions are indicated by corresponding abbreviations on days when they were performed. BP: blood pressure measurement, YM: Y-maze alternation test, CBF: cerebral blood flow measurement, T: treatment.*

Y-maze alternation test

The test was performed the same way as described in Exp 1. on Treatment Day 8 (*see* Figure S2).

Blood pressure measurement

Blood pressures were measured as described in Exp 1 with an additional refinement measure: cream cheese, also after being introduced to the animals, was applied to the outer rim of the "head-holding" cone. By getting them familiar with the set-up (and the cheese as an “if-necessary” motivator), we had a greater chance to measure the animals in a relatively calmer state by the time of the experiment itself. After 3 habituation measurements within a week preceding the treatment period, baseline was recorded on Treatment Day 1 prior to the first injection. Further measurements were carried out on Day 2, 5 and 8 (*see* Figure S2).

CBF was measured in the same mode as described in Exp1. on Day 9.

Tissue collection and sampling

Brain hemispheres (excluding the cerebellum), upper part of small intestine and kidneys were collected after the CBF measurements.

Tissue samples for ELISA tests were preserved the same way as detailed in Exp 1.

ELISA measurements for EET levels and kidney tissue staining (6 samples from each group) were done as detailed in Exp 1 and 2., respectively.

**Exp 4:** *Conditioned Taste Aversion test with the cocktail in aged rats*

Conditioned taste aversion with LiCl

We conducted this study with 12 naïve male Hannover Wistar rats (Toxicoop, Budapest, Hungary) weighing 240-260 g and being 2-2.5 months of age at the start of the experiment.

On Day 1, water was withdrawn from the group-housed animals for a 20-hour water deprivation period. On Day 2, animals were separated into individual cages, and two water bottles were introduced (for 4 hours) to measure baseline water consumption and to assess drinking patterns for potential side preferences. Day 3 involved taste aversion conditioning: animals got access to two bottles containing 0.3% saccharin solution. An hour later the animals were IP administered 1.2 ml LiCl solution (30 mg/ml, Reanal Laboratory Chemicals Ltd., Budapest, Hungary) or saline. Afterwards, the saccharin bottles were available for an additional hour. On Days 4 and 5, animals were presented with a saccharin solution-containing and a water-containing bottle. Fluid consumption was measured for four hours to determine saccharin preference *(=saccharin solution consumption / (saccharin solution + water consumption) x 100*).

**Results**

**Exp 1:** *Dose finding in young naïve Wistar rats*

There was no significant difference between groups in the mean change of CBF in the barrel cortex in response to the stimulation of the contralateral whisker pad (Figure S3). We also did not see a significant difference in the systolic or the diastolic blood pressure in the two groups although an overall tendency of increase can be seen in the cocktail treated group. This group also showed a significantly lower heart rate compared to controls (F(1, 15)=13.24, p < 0.005). Post-hoc analysis (Duncan test) revealed a significant difference between the two groups on Day 2 (p < 0.05). There was no significant difference in EET levels neither in hippocampus nor in barrel-cortex samples. No significant difference was observed in the Y-maze test. Unfortunately, very few animals produced valid data in the lever press and NOR assays, so the outcome of these tests was not evaluable.

*Fig. S3 – Main results of Exp 1.* ***a)*** *increase in CBF in the barrel cortex in response to whisker stimulation (mean ± SEM.* ***b)*** *percent of alternations in the Y-maze* ***c) and d)*** *systolic blood pressure and heart rate, respectively, over the course of vehicle or cocktail treatment (mean ± SEM). *: p < 0.05 vs ’vehicle’, post-hoc Duncan test after repeated measures ANOVA.* ***e) and f)*** *11,12-EET levels in the barrel cortex and hippocampus (respectively) (mean ± SEM).*

**Exp 2** *Cognitive effects of the uncoupling cocktail in aged, experienced Long-Evans rats*

***Table S2*** *Scoring of alterations caused by treatment in kidney tissue sections. Assigned score for each category correspond to the following: 0 = No pathological changes, 1 = Minimal changes (affecting ≤10% of the section), 2 = Mild changes (affecting 11–25% of the section), 3 = Moderate changes (affecting 26–50% of the section), 4 = Severe changes (affecting >50% of the section).*

| vehicle-treated | animal ID | | | | | | | | | | | |
| --- | --- | --- | --- | --- | --- | --- | --- | --- | --- | --- | --- | --- |
| Alteration | 1 | 1C | C | 11 | 20 | E | 2 | 4 | 5 | 6 | A | 15 |
| Vacuolization of tubular epithelium (focal) | 0 | 1 | 0 | 1 | 0 | 0 | 1 | 1 | 1 | 0 | 1 | 1 |
| Vacuolization of tubular epithelium (multifocal or diffuse) | 0 | 0 | 0 | 0 | 0 | 2 | 0 | 0 | 0 | 0 | 0 | 0 |
| Tubular dilatation | 0 | 0 | 0 | 0 | 0 | 0 | 0 | 0 | 0 | 1 | 1 | 0 |
| Eosinophil casts in the tubular lumen | 0 | 0 | 0 | 0 | 0 | 0 | 0 | 0 | 0 | 0 | 0 | 0 |
| Brown pigments in the tubular epithelium | 0 | 0 | 0 | 0 | 0 | 1 | 2 | 2 | 1 | 1 | 1 | 1 |
| Apoptosis in the tubular epithelium | 0 | 0 | 0 | 0 | 0 | 0 | 0 | 0 | 0 | 0 | 0 | 0 |
| Apoptosis in the medulla | 0 | 0 | 0 | 0 | 0 | 0 | 0 | 0 | 0 | 0 | 0 | 0 |
| Total score | 0 | 1 | 0 | 1 | 0 | 3 | 3 | 3 | 2 | 2 | 3 | 2 |
| cocktail-treated | animal ID | | | | | | | | | | |  |
| Alteration | 0 | 8 | 12 | 13 | 14 | 1A | 21 | 3 | 19 | 1F | 1E |  |
| Vacuolization of tubular epithelium (focal) | 0 | 0 | 0 | 0 | 0 | 0 | 0 | 0 | 0 | 0 | 0 |  |
| Vacuolization of tubular epithelium (multifocal or diffuse) | 3 | 3 | 3 | 2 | 2 | 2 | 0 | 3 | 2 | 2 | 2 |  |
| Tubular dilatation | 2 | 2 | 1 | 4 | 3 | 2 | 2 | 3 | 3 | 2 | 2 |  |
| Eosinophil casts in the tubular lumen | 2 | 2 | 1 | 3 | 2 | 2 | 1 | 1 | 1 | 2 | 2 |  |
| Brown pigments in the tubular epithelium | 3 | 3 | 3 | 3 | 2 | 2 | 0 | 0 | 1 | 0 | 0 |  |
| Apoptosis in the tubular epithelium | 1 | 3 | 1 | 1 | 1 | 1 | 0 | 0 | 1 | 1 | 1 |  |
| Apoptosis in the medulla | 0 | 2 | 3 | 2 | 1 | 3 | 1 | 1 | 1 | 2 | 2 |  |
| Total score | 11 | 15 | 12 | 15 | 11 | 12 | 4 | 8 | 9 | 9 | 9 |  |

**Exp 3**: *Effects of MS-PPOH in young naïve rats*

The MS-PPOH-treated group exhibited a greater mean change from baseline in CBF compared to controls due to two outlier values, however, this difference was not statistically significant (Figure S4).

No significant effects were observed for either systolic blood pressure or heart rate across measurements. However, a significant time effect was revealed on diastolic blood pressure (time: F(3, 39)=2.941, p < 0.05.).

*Fig.S4. Results of Exp 3 –* ***a)*** *increase in CBF in the barrel cortex in response to whisker stimulation (mean ± SEM).* ***b) and c)*** *Number of entries and percent of alternations in the Y-maze, respectively (mean ± SEM).* ***d), e) and f)*** *systolic, diastolic blood pressure and heart rate, respectively, over the course of vehicle or cocktail treatment (mean ± SEM).* ***g) and h)*** 11,12-EET levels in the brain and kidney, respectively (mean ± SEM). *: p < 0.05, t*-test.*

Y-maze performance analysis revealed no significant between-group difference in spontaneous alternation behaviour.

EET levels in renal tissue showed a statistically significant difference between groups (t(13)=2.849, p < 0.05). No other measured analytes demonstrated significant variations.

We did not observe notable histological alterations in kidney sections (mean histology scores of the vehicle-treated and cocktail-treated groups were 1.2±0.48 and 0.33±0.21, respectively; p>0.05, Mann-Whitney U-test).

**Exp 4***: Conditioned taste aversion test with the cocktail in aged rats*

*Conditioned taste aversion test with LiCl*

After conditioning, during experimental days 4 and 5 when animals had simultaneous access to water and saccharin solution, animals treated with LiCl showed significantly reduced saccharin preference compared to controls (Day 4: t(10) = 19.905, p < 0.001; Day 5: t(10) = 29.651, p < 0.001).

*Fig. S5 – Investigation of CTA evoked by LiCl - Proportion of saccharin solution consumption in a two-bottle choice situation (saccharin solution and water) after pairing saccharin consumption with LiCl or saline treatment (mean ± SEM). The dotted line marks the ‘no-preference’ value. *****: p < 0.001, vs ‘Saline’ t-test.*
